# Progress, challenge and prospect of plant plastome annotation

**DOI:** 10.3389/fpls.2023.1166140

**Published:** 2023-05-30

**Authors:** Xiao-Jian Qu, Dan Zou, Rui-Yu Zhang, Gregory W. Stull, Ting-Shuang Yi

**Affiliations:** ^1^ Shandong Provincial Key Laboratory of Plant Stress Research, College of Life Sciences, Shandong Normal University, Ji’nan, Shandong, China; ^2^ Germplasm Bank of Wild Species, Kunming Institute of Botany, Chinese Academy of Sciences, Kunming, Yunnan, China; ^3^ University of Chinese Academy of Sciences, Beijing, China

**Keywords:** annotation standards, chloroplast genome, plastome, protein structure, pseudogenes, RNA-editing genes

## Abstract

The plastome (plastid genome) represents an indispensable molecular data source for studying phylogeny and evolution in plants. Although the plastome size is much smaller than that of nuclear genome, and multiple plastome annotation tools have been specifically developed, accurate annotation of plastomes is still a challenging task. Different plastome annotation tools apply different principles and workflows, and annotation errors frequently occur in published plastomes and those issued in GenBank. It is therefore timely to compare available annotation tools and establish standards for plastome annotation. In this review, we review the basic characteristics of plastomes, trends in the publication of new plastomes, the annotation principles and application of major plastome annotation tools, and common errors in plastome annotation. We propose possible methods to judge pseudogenes and RNA-editing genes, jointly consider sequence similarity, customed algorithms, conserved domain or protein structure. We also propose the necessity of establishing a database of reference plastomes with standardized annotations, and put forward a set of quantitative standards for evaluating plastome annotation quality for the scientific community. In addition, we discuss how to generate standardized GenBank annotation flatfiles for submission and downstream analysis. Finally, we prospect future technologies for plastome annotation integrating plastome annotation approaches with diverse evidences and algorithms of nuclear genome annotation tools. This review will help researchers more efficiently use available tools to achieve high-quality plastome annotation, and promote the process of standardized annotation of the plastome.

## 1 Introduction

The endosymbiotic evolutionary origin of mitochondria and plastids was proposed more than a century ago. Konstantin Mereschkowsky was considered the first person to explore the similarities between “*Cyanophyceae*” (cyanobacteria) and the “chromatophores” (chloroplasts/plastids) of plants (Mereschkowsky, 1905; Martin and Kowallik, 1999). The symbiogenesis concept proposed by Mereschkowsky was advocated by Margulis (1970), and the endosymbiosis hypothesis has been widely accepted by the scientific community. Plastid genomes (plastomes) contain up to ~250 genes (Hagopian et al., 2004), while at least 1700 genes are present in cyanobacterial genomes (Rocap et al., 2003). Following the transition from free-living bacteria to plastids, the plastome has significantly reduced its gene content and size.

**Figure 1:**
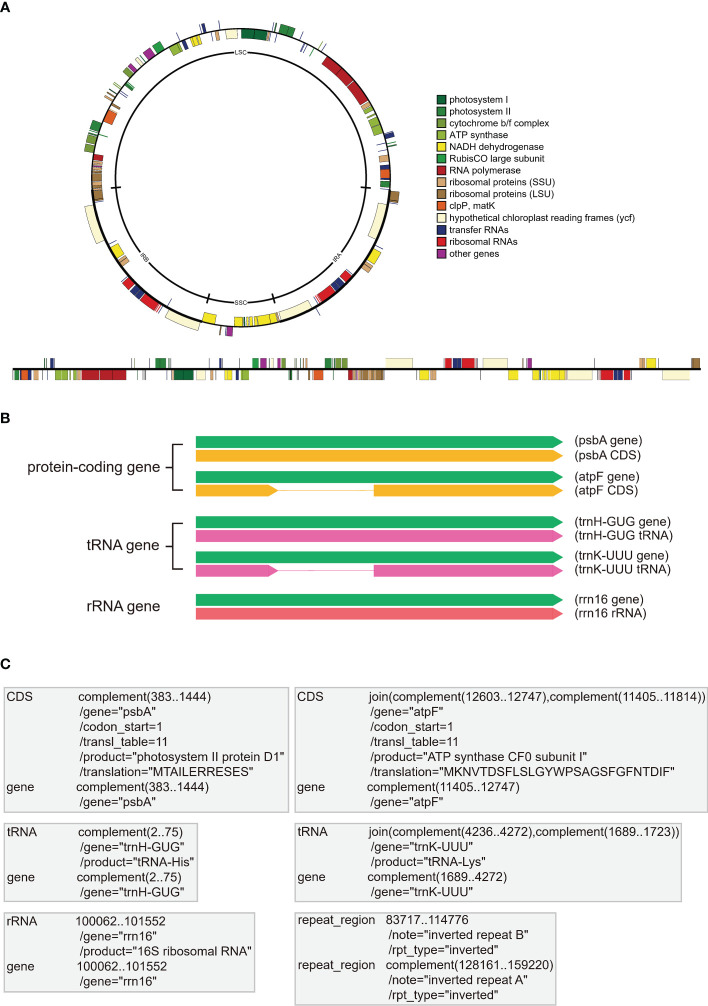
The display form and storage form of annotation information in plastomes. **(A)** The common quadripartite structure of plastomes with circular and linear forms. All plastome genes are categorized into different color bars with different functions. **(B)** The display forms of three gene types in plastomes, including PCGs (e.g., *psbA* and *atpF*), tRNAs (e.g., *trnH-GUG* and *trnK-UUU*), and rRNAs (e.g., *rrn16*). The green bars indicate the essential gene element for all three gene types, the yellow bars indicate the coding sequences (CDS) of PCGs without or with introns, the pink bars indicate the coding sequences of tRNAs without or with introns, the red bar indicates the coding sequences of rRNA. **(C)** The essential “FEATURES” and “Location/Qualifiers” information for the three gene types (i.e., PCGs, tRNAs, and rRNAs) in GenBank flatfiles. The first column shows the “FEATURES” of PCGs (e.g., *psbA* and *atpF*), tRNAs (e.g., *trnH-GUG* and *trnK-UUU*), rRNAs (e.g., *rrn16*) and IRb, and the second column shows the “Location/Qualifiers” of PCGs, tRNAs, rRNAs and IRb, where the coordinates shown as numbers are “Location” elements and the lines start with the “/” symbol (e.g.,/gene and/product) indicate the “Qualifiers” elements.

The plastomes of most photosynthetic seed plants are highly conserved, with a quadripartite structure comprising large (LSC) and small (SSC) single-copy regions and two inverted repeat (IR) regions (**Figure 1A**; Wicke et al., 2011; Jansen and Ruhlman, 2012). The plastomes of photosynthetic seed plants are usually 120-160 kb and contain 101-118 unique genes, including ~80 protein-coding genes (PCGs), ~30 transfer RNA genes (tRNAs), and four ribosomal RNA genes (rRNAs) (**Figure 1B**, Jansen and Ruhlman, 2012). Plastid genes generally fall into the following categories: NADH complex genes (*ndhA-K*), PEP genes (*rpoA*/*B*/*C1*/*C2*), photosynthesis genes (*pet*, *psa*, *psb*, *ccsA*, *cemA*, *ycf3*, *ycf4*), *rbcL*, ATP synthesis (*atp*) genes, translation apparatus genes (ribosomal protein subunits: *rpl*, *rps*, *matK*, *infA*; rDNAs: *rrn*; tRNAs: *trn*), and other genes (e.g., *accD*, *clpP*, *ycf1*, and *ycf2*) (**Figure 1A**; **Table 1**). The coding sequence (CDS) need to meet the criterion of correct open reading frame (orf), the tRNAs need to meet the criterion of cloverleaf structure, and the rRNAs need to meet the criterion of high sequence similarity with reference species.

**Table 1:** Gene names and corresponding products in plastomes of seed plants.

Gene	Product	Gene	Product
*accD*	acetyl-CoA carboxylase carboxyltransferase beta subunit	*rpl32*	ribosomal protein L32
*atpA*	ATP synthase CF1 alpha subunit	*rpl33*	ribosomal protein L33
*atpB*	ATP synthase CF1 beta subunit	*rpl36*	ribosomal protein L36
*atpE*	ATP synthase CF1 epsilon subunit	*rpoA*	RNA polymerase alpha subunit
*atpF*	ATP synthase CF0 subunit I	*rpoB*	RNA polymerase beta subunit
*atpH*	ATP synthase CF0 subunit III	*rpoC1*	RNA polymerase beta’ subunit
*atpI*	ATP synthase CF0 subunit IV	*rpoC2*	RNA polymerase beta’’ subunit
*ccsA*	cytochrome c heme attachment protein	*rps11*	ribosomal protein S11
*cemA*	envelope membrane protein	*rps12*	ribosomal protein S12
*chlB*	photochlorophyllide reductase subunit B	*rps14*	ribosomal protein S14
*chlL*	photochlorophyllide reductase subunit L	*rps15*	ribosomal protein S15
*chlN*	photochlorophyllide reductase subunit N	*rps16*	ribosomal protein S16
*clpP*	clp protease proteolytic subunit	*rps18*	ribosomal protein S18
*infA*	translation initiation factor 1	*rps19*	ribosomal protein S19
*matK*	maturase K	*rps2*	ribosomal protein S2
*ndhA*	NADH-plastoquinone oxidoreductase subunit 1	*rps3*	ribosomal protein S3
*ndhB*	NADH-plastoquinone oxidoreductase subunit 2	*rps4*	ribosomal protein S4
*ndhC*	NADH-plastoquinone oxidoreductase subunit 3	*rps7*	ribosomal protein S7
*ndhD*	NADH-plastoquinone oxidoreductase subunit 4	*rps8*	ribosomal protein S8
*ndhE*	NADH-plastoquinone oxidoreductase subunit 4L	*ycf1*	hypothetical protein RF1
*ndhF*	NADH-plastoquinone oxidoreductase subunit 5	*ycf2*	hypothetical protein RF2
*ndhG*	NADH-plastoquinone oxidoreductase subunit 6	*ycf3*	photosystem I assembly protein Ycf3
*ndhH*	NADH-plastoquinone oxidoreductase subunit 7	*ycf4*	photosystem I assembly protein Ycf4
*ndhI*	NADH-plastoquinone oxidoreductase subunit I	*trnA-UGC*	tRNA-Ala
*ndhJ*	NADH-plastoquinone oxidoreductase subunit J	*trnC-GCA*	tRNA-Cys
*ndhK*	NADH-plastoquinone oxidoreductase subunit K	*trnD-GUC*	tRNA-Asp
*petA*	cytochrome f	*trnE-UUC*	tRNA-Glu
*petB*	cytochrome b6	*trnF-GAA*	tRNA-Phe
*petD*	cytochrome b6/f complex subunit IV	*trnfM-CAU*	tRNA-Met
*petG*	cytochrome b6/f complex subunit V	*trnG-GCC*	tRNA-Gly
*petL*	cytochrome b6/f complex subunit VI	*trnG-UCC*	tRNA-Gly
*petN*	cytochrome b6/f complex subunit VIII	*trnH-GUG*	tRNA-His
*psaA*	photosystem I P700 apoprotein A1	*trnI-CAU*	tRNA-Ile
*psaB*	photosystem I P700 apoprotein A2	*trnI-GAU*	tRNA-Ile
*psaC*	photosystem I subunit VII	*trnK-UUU*	tRNA-Lys
*psaI*	photosystem I subunit VIII	*trnL-CAA*	tRNA-Leu
*psaJ*	photosystem I subunit IX	*trnL-UAA*	tRNA-Leu
*psaM*	photosystem I protein M	*trnL-UAG*	tRNA-Leu
*psbA*	photosystem II protein D1	*trnM-CAU*	tRNA-Met
*psbB*	photosystem II CP47 chlorophyll apoprotein	*trnN-GUU*	tRNA-Asn
*psbC*	photosystem II CP43 chlorophyll apoprotein	*trnP-GGG*	tRNA-Pro
*psbD*	photosystem II protein D2	*trnP-UGG*	tRNA-Pro
*psbE*	photosystem II cytochrome b559 alpha subunit	*trnQ-UUG*	tRNA-Gln
*psbF*	photosystem II cytochrome b559 beta subunit	*trnR-ACG*	tRNA-Arg
*psbH*	photosystem II phosphoprotein	*trnR-CCG*	tRNA-Arg
*psbI*	photosystem II protein I	*trnR-UCU*	tRNA-Arg
*psbJ*	photosystem II protein J	*trnS-GCU*	tRNA-Ser
*psbK*	photosystem II protein K	*trnS-GGA*	tRNA-Ser
*psbL*	photosystem II protein L	*trnS-UGA*	tRNA-Ser
*psbM*	photosystem II protein M	*trnT-GGU*	tRNA-Thr
*psbN*	photosystem II protein N	*trnT-UGU*	tRNA-Thr
*psbT*	photosystem II protein T	*trnV-GAC*	tRNA-Val
*psbZ*	photosystem II protein Z	*trnV-UAC*	tRNA-Val
*rbcL*	ribulose bisophosphate carboxylase	*trnW-CCA*	tRNA-Trp
*rpl14*	ribosomal protein L14	*trnY-GUA*	tRNA-Tyr
*rpl16*	50S ribosomal protein L16	*rrn16*	16S ribosomal RNA
*rpl2*	ribosomal protein L2	*rrn23*	23S ribosomal RNA
*rpl20*	ribosomal protein L20	*rrn4.5*	4.5S ribosomal RNA
*rpl22*	ribosomal protein L22	*rrn5*	5S ribosomal RNA
*rpl23*	ribosomal protein L23		

**Table 2:** Example comparison of gene numbers for assessing plastome annotation quality.

H:hitting, M:missing, N:total, n:number, p:percentage, P:PCGs, T:tRNAs, R:rRNAs
1. Number of hitting and missing genes:
|112 number of hitting genes (nH)|
|2 number of missing genes (nM)|
|98.25% percentage of hitting gene (pH)|
|1.75% percentage of missing genes (pM)|
|114 total gene numbers (N)|
2. Number of hitting and missing PCGs, tRNAs and rRNAs:
|78 number of hitting PCGs (nHP)|
|2 number of missing PCGs (nMP)|
|30 number of hitting tRNAs (nHT)|
|0 number of missing tRNAs (nMT)|
|4 number of hitting rRNAs (nHR)|
|0 number of missing rRNAs (nMR)|
3. Name of hitting and missing PCGs, tRNAs and rRNAs:
|name of hitting PCGs|
*psbA*, *matK*, …
|name of missing PCGs|
*infA*, *ccsA*, …
|name of hitting tRNAs|
*trnA-UGC*, …
|name of missing tRNAs|
None
|name of hitting rRNAs|
*rrn16*, *rrn23*, …
|name of missing rRNAs|
None

The GenBank format flatfile, which can be easily downloaded from NCBI, is widely used to store annotation information. As one of the most common formats, GenBank flatfile contains an annotation section and a sequence section (Calabrese, 2018). This GenBank flatfile is always suffixed with “.gb” or “.gbk”. With regards to plastome annotation, the annotation section of GenBank flatfile, between the line starting with the “FEATURES” element and the line starting with “ORIGIN” element, contains the most important information for accurate annotation. The official guidance note (Calabrese, 2018) explained “FEATURES” as “Information about genes and gene products, as well as regions of biological significance reported in the sequence. These can include regions of the sequence that code for proteins and RNA molecules, as well as a number of other features”. In **Figure 1C**, we briefly show the essential information for the three gene types, PCGs, tRNAs, and rRNAs. The “gene” FEATURE is essential for each gene, including PCGs, tRNAs, and rRNAs, and is followed by the Location of its nucleotides in the plastome. The “/gene” Qualifier is placed in the next row being assigned the gene name. The “CDS” FEATURE is exclusive for PCGs and is followed by the Location of its nucleotides from start codon to stop codon that corresponds with the amino acid sequences of a protein. The “/gene” Qualifier is also placed in the next row being assigned the gene name. The “/codon_start”, “/transl_table”, “/product” and “/translation” Qualifiers appear successively, with the first two as a default set for plastome and the last two indicating the translation product (see **Table 1**) and the amino acid translation of the gene, respectively. The “tRNA” and “rRNA” FEATURES are exclusive for tRNA and rRNA genes, respectively and are followed by the Location of its nucleotides in the plastome. The “/gene” Qualifier is also placed in the next row being assigned the gene name. The “/product” Qualifier appears in the next row, indicating the expression product of the gene. Furthermore, people can annotate the region of IRb and IRa as the “repeat_region” FEATURE and the “/note” and “/rpt_type” Qualifiers (**Figure 1C**). In addition, other FEATURES and Location/Qualifiers that meet the requirement of GenBank flatfile can be added as well.

Newly developed sequencing technologies and assembly and annotation tools have jointly contributed to a rapid increase in the number of complete plastome. The genome skimming approach, in particular, has been widely used for plastome sequencing, given that plastid DNA is over-represented in genomic DNA due to its high-copy nature (Straub et al., 2012). The assembly of plastome sequences can be performed using *de novo* assemblers, e.g., SOAPdenovo2 (Luo et al., 2012) and Spades (Bankevich et al., 2012), or specialized organelle or plastome assemblers, e.g., NOVOplasty (Dierckxsens et al., 2017) or GetOrganelle (Jin et al., 2020). Considering the special quadripartite structure of the plastome, an automated solution for plastome assembly and structure standardization, NOVOWrap, was proposed (Wu et al., 2021). Due to the peculiarities of the plastome, i.e. circular structure, small genome size, and dense gene distribution, it is usually annotated using specialized tools, e.g., the web tool GeSeq (Tillich et al., 2017) or the command-line tool PGA (Qu et al., 2019). Plastaumatic, an automated pipeline for both assembly and annotation of plastomes, was recently developed (Chen et al., 2022).

**Figure 2:**
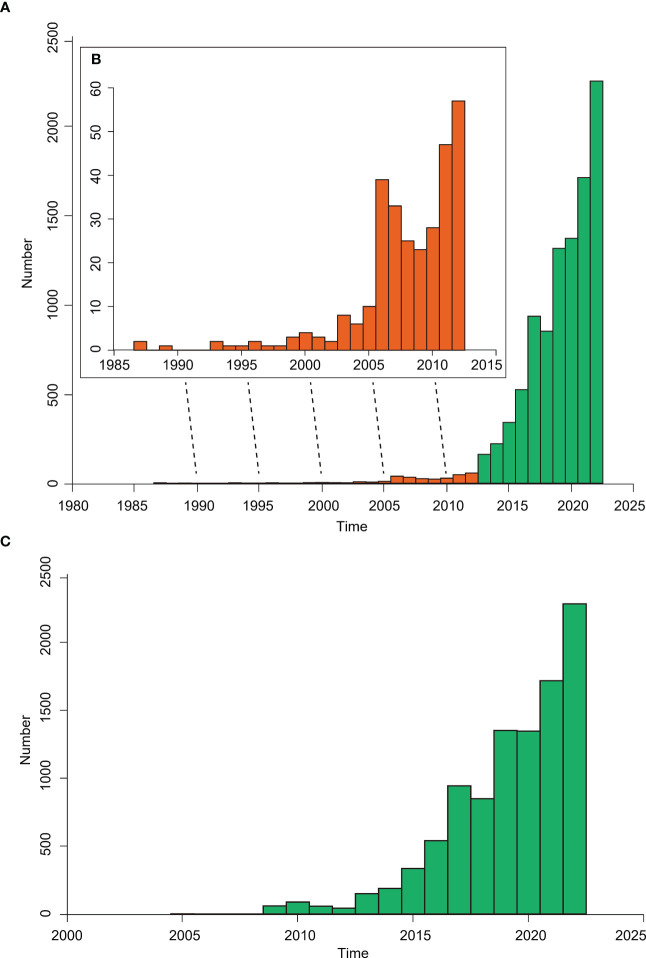
The bar chart showing the plastome numbers of green plants released in the Organelle Genome Resources of GenBank. **(A)** The number of reported complete plastomes per year from 1986 to 2022, with the bars from 1986 to 2012 shown as orange color and the bars from 2013 to 2022 shown as green color. **(B)** The inset graph shows the enlargement of bars from 1986 to 2012. **(C)** The release number of plastomes that meet the RefSeq standard defined by GenBank from 2004 to 2022.

As of September 23, 2022, a total of 9,951 plastome sequences of green plants are available on the Organelle Genome Resources of GenBank (https://www.ncbi.nlm.nih.gov/genome/organelle/; **Figure 2A**). From 1986 to 2005, the number of reported complete plastomes were ≤10 each year, and a total of 47 plastomes were published during those twenty years (**Figure 2A**). From 2006 to 2012, a total of 252 plastomes were reported with 23-57 plastomes per year (**Figure 2A**). From 1986 to 2012, a total of 299 plastomes were reported. From 2013 to Sep 23, 2022, the number of reported plastomes increase rapidly ranging from 161 to 2245, and a total of 9652 plastomes were reported (**Figure 2A**). In addition, the number of plastomes that meet the RefSeq standard defined by GenBank is also increasing rapidly (**Figure 2C**). From 2009 to 2012, a total of 247 RefSeq plastomes were released with 43-88 plastomes per year. From 2013 to Sep 23, 2022, a total of 9702 plastomes were released, and the number of plastomes released each year increased rapidly from 150 to 2283 (**Figure 2C**).

These plastome resources have been applied in diverse areas of the plant sciences including phylogenomics, evolutionary biology, comparative genomics, population genomics, phylogeography, chloroplast genetic engineering, and so on (Daniell et al., 2021). However, researchers often meet some common problems that are related to the accuracy of plastome assembly and annotation. Because assembly is an upstream step of annotation, assembly quality will directly influence the accuracy of plastome annotation. Therefore, if we want to obtain high-quality plastome annotations, we need to ensure that the assembled plastome sequence is reliable. Annotation quality greatly affects the downstream plastome applications, such as phylogenetic tree construction, comparative genomics, and chloroplast genetic engineering. The NCBI website states that the organelle genomes with curated annotation information from the Reference Sequence (RefSeq) project can be used as the standard. However, although the GenBank plastome sequences have met RefSeq standards, there are still some annotation complications and even errors.

Frequently used plastome annotation tools include DOGMA (Wyman et al., 2004), CpGAVAS (Liu et al., 2012), CGAP (Cheng et al., 2013), ORG.Annotate (https://git.metabarcoding.org/org-asm/org-annotate), Plann (Huang and Cronk, 2015), Verdant (McKain et al., 2017), GeSeq (Tillich et al., 2017), AGORA (Jung et al., 2018), CpGAVAS2 (Shi et al., 2019), PGA (Qu et al., 2019), and Chloe (https://chloe.plastid.org/annotate.html). Guyeux et al. (2019) compared the two main plastome annotation tools that were available by 2019, GeSeq and Dogma, and found more consistent annotation of genes by GeSeq. However, there is no comprehensive comparison of all released annotation tools. Furthermore, none of the existing plastome annotation tools can produce annotations suitable for direct submission to GenBank without manual correction. The major problems are gene annotation errors and the absence of a standard GenBank annotation flatfile, which are mainly caused by the use of different annotation tools with different annotation strategies and different reference plastomes, and by users employing their own standards for correct plastome annotations. In this review, we compare the annotation principles of commonly used plastome annotation tools, show common annotation errors and provide relevant solutions, propose the necessity of establishing a database of reference plastomes with standardized annotations, put forward quantitative standards for high-quality plastome annotations, and discuss how to generate standard GenBank annotation flatfiles for submission and downstream operations.

## 2 Challenge and relevant solutions

### 2.1 Comparison of principles of plastome annotation tools

#### 2.1.1 Introduction of genome annotation principle

Assembled genome sequences alone have limited biological significance; genome annotations are important for adding meaningful biological information to genome sequences. Therefore, genome annotation, including structural and functional annotations, is an indispensable step in genomic studies.

Structural annotation is the process of locating gene and its introns. In other words, structural annotation is mainly about gene prediction, including *ab initio* gene prediction and homology-based gene prediction. The *ab initio* gene prediction method is a complex process that depends not only on the open reading frame (ORF), but also on the frequency of codon usage and GC content, especially for intron-containing genes. The *ab initio* gene prediction method is mainly applied to nuclear genomes with complex gene structures; it is rarely applied to plastomes with conserved gene numbers, gene structures, and dense gene distributions. The homology-based method is widely used in gene prediction, and related evidence includes homologous (to be exact, orthologous) gene sequences of reference species, RNA-seq reads, cDNA sequences, and protein sequences. For plastome annotation, homology-based gene prediction is the mainstream approach, mainly due to the conserved gene numbers, structures, sequence similarity, and the near-absence of paralogous genes in plastomes. At present, plastome gene prediction largely depends on homologous gene sequences of closely-related reference species. When cDNA sequences or protein sequences are available, exon-intron boundaries are relatively easy to infer, but such references are not always available.

Functional annotation is the process of associating biological information with the genes after structural annotation. The homology-based method is most widely applied, which involves searching for highly similar protein sequences of predicted genes from protein databases. Domain regions and gene ontology terms can be obtained through the specific tools. For plastomes, when we complete homology-based structural annotation, the corresponding products of PCGs, tRNAs and rRNAs can be easily linked (**Table 1**), which is due to the limited gene numbers of plastomes.

#### 2.1.2 Comparison of the differences and similarities between plastome and nuclear genome annotation

The ultimate goal of both plastome and nuclear genome annotation is to identify all types of functional elements and their occurrences in the genome. Both processes face difficulties of annotating pseudogenes, which are common in eukaryotic nuclear genomes and plastomes of heterotrophic plants, but rare in plastomes of autotrophic plants. Both processes need to treat RNA-editing events, which occur in both nuclear and plastid genomes. However, annotation of nuclear genomes is much more difficult than annotation of plastomes for the following five reasons. First, the nuclear genome has many more genes (tens of thousands) than do plastomes (over one hundred), and plastomes of autotrophic plants have conserved genes and gene numbers. In addition, the dense distribution of plastid genes and relatively short intergenic stretches between every pair of genes makes it almost impossible to discover new genes. Second, non-coding RNAs of nuclear genomes usually include tRNAs, rRNAs, miRNAs, and snRNAs, and regulatory regions of nuclear genomes usually include promoters, enhancers, silencers, and insulators. In contrast, only tRNAs and rRNAs need to be annotated for plastomes. Third, genes in nuclear genome show high number variation for their introns, and alternative splicing is much common for nuclear genes with many exons. However, plastid genes usually do not have introns; rarely they have one or two, and the only three plastid genes contain two introns: *rps12*, *clpP* and *ycf3*. Fourth, nuclear genomes usually have a large number of structural variations, and segmental duplication results in many paralogous genes. For nuclear genomes, similarities to proteins in other species might suffer from the orthologue-paralogue problem. However, only two IR copies of plastome have completely identical genes, and a few plastid genes have duplicated copies in the single-copy regions. Fifth, repetitive, or interspersed, elements are an important feature of eukaryotic nuclear genomes and account for a large proportion of the variation in genome size. However, the plastome size is small, and repetitive elements, especially long repetitive elements, are rare in plastome. Therefore, nuclear genome annonation requires a variety of different strategies compared to plastome annotation (Salzberg, 2019; Ejigu and Jung, 2020).

#### 2.1.3 Comparison of principles of major available plastome annotation tools

DOGMA, a widely used web tool for annotating organellar genomes (Wyman et al., 2004), locates plastid genes by using BLAST to search against a database including 16 plastomes of green plants. To annotate PCGs, the target plastome is translated into all six open reading frames, which are searched against amino acid references in the database; to annotate tRNAs and rRNAs, the nucleotides in the target plastome are searched against nucleotide references in the database. However, DOGMA has two major limitations. One, DOGMA cannot annotate plastomes in batch mode. Second, DOGMA can only locate the approximate regions of the target genes *via* BLAST, so start codons and stop codons, exon-intron boundaries, and the boundaries of tRNAs and rRNAs should be manually defined.

CPGAVAS is an integrated web tool for plastome annotation, visualization, and analysis (Liu et al., 2012). It predicts PCGs and rRNAs by identifying and mapping the most similar full-length protein, cDNA, and rRNA sequences on the target plastome sequences by integrating the results from BLASTX, BLASTN, protein2genome, and est2genome. It predicts tRNAs and IRs using tRNAscan and ARAGORN, and vmatch, respectively. CPGAVAS2 is a significantly updated version that includes a 43-plastome reference dataset curated based on RNA-seq data, and two new algorithms for annotating small exons and the trans-splicing gene *rps12* (Shi et al., 2019). However, this tool cannot conduct annotation of a large number of plastome sequences at one time, and manual correction is usually needed.

GeSeq was designed for the rapid and accurate annotation of plant organelle genome sequences, particular for plastomes (Tillich et al., 2017). GeSeq provides an integrated database with manually curated reference sequences. The genes can be identified by BLAT-based homology searches, using profile HMM searches for PCGs and rRNAs, and tRNAscan-SE and ARAGORN for tRNAs. It can not only annotate plastomes using batch mode, but also includes flexibility for selecting reference plastomes, including those selected from NCBI or uploaded by users. However, GeSeq has some limitations also shared by DOGMA and CPGAVAS—for example, there is no specific algorithm for detecting extremely short exons.

PGA is a command-line tool that can perform rapid, accurate, and flexible batch annotation of plastomes (Qu et al., 2019). In contrast to other existing tools, PGA uses reference plastomes as the query and unannotated target plastomes as the subject to locate genes, i.e., the reverse query-subject BLAST search approach. Specifically, both BLASTN and TBLASTN searches are conducted for PCGs. In order to refine gene and exon-intron boundaries initially determined with BLAST searches, two boundary detection algorithms were developed. For PCGs with short first exons, it uses the short exon sequence as a probe to search the region between the 5’-end of the much longer exon and the first detected upstream gene. In addition, intron loss events can be detected. Users can select the built-in reference plastomes or flexibly select their own uploaded reference plastomes. A parameter (-q or -qcoverage) with two values to judge pseudogenes is provided. If the annotated genes have a query coverage less or greater than each of these two values, they are not removed from the annotations but the related warning information is recorded in the log file allowing the user to determine whether the genes are pseudogenes or redundant false-positive gene fragments. Therefore, several gene fragments annotated by PGA may be redundant, because PGA cannot distinguish false-positive gene fragments from pseudogenes. However, this feature of PGA may be important for searching the pseudogene residues, which are important remnants for tracing the evolutionary history of these fast-evolving genes, especially in heterotrophic plants with highly degraded plastomes.

Chloe can predict genes by transferring high-quality, manually-curated annotations of a few model plastomes to other plastomes; this is achieved by aligning whole plastome sequences, projecting the reference features to corresponding syntenic regions, and interpreting the projected features with all available biological information (Zhong, 2020). The input files include a set of reference plastomes with annotations, the unannotated target plastome sequences, and a feature template, which can be manually adjusted depending on the degree of conservation of coding sequences and introns. In the gene model construction step, the RNA-editing events are considered when determining the start codons and stop codons, and the annotation accuracy of gene and exon-intron boundaries is highly dependent on the annotation quality of designated reference plastomes. Chloe can also generate a number of redundant false-positive gene fragments that may be pseudogene remnants of highly divergent genes. In addition, some PCGs with short exons and the trans-splicing gene *rps12* also need manual confirmation. As mentioned by the authors, a final correction is still needed, especially for heterotrophic plants.

In short, none of the existing plastome annotation tools can produce annotations suitable for direct submission to GenBank without further manual confirmation. One issue requiring special attention is the annotation quality of reference plastomes, which has a significant impact on the annotation accuracy of all above-mentioned tools. Indeed, the improved annotation quality of newly developed tools is largely dependent on the use of high-quality reference plastomes. The major improvements of the newly released tools largely pertain to special methods for specific genes, such as highly divergent genes, pseudogenes, trans-splicing genes, genes with short exons, duplicated genes, and RNA-editing genes. However, none of these tools is able to deal with all of these special cases. In addition, the majority of plastome annotation tools only provide a web service, which is tedious and time-consuming when annotating hundreds or thousands of plastome sequences. Future plastome annotation tools should attempt to address these shortcomings.

#### 2.1.4 Application of plastome annotation tools

**Figure 3:**
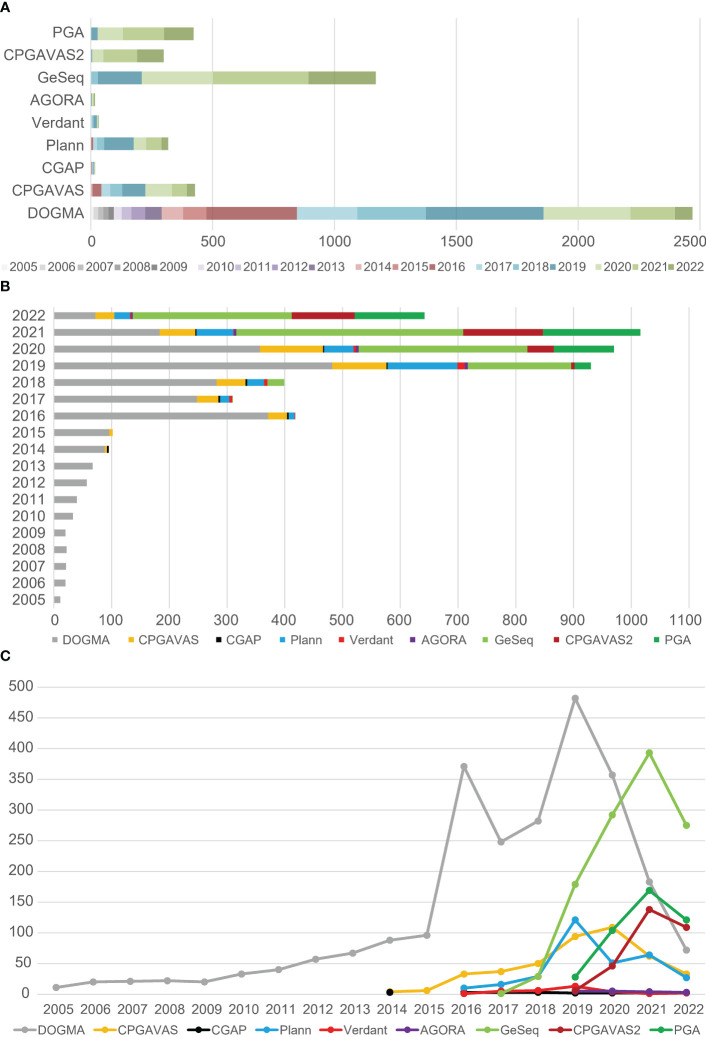
The citation number of currently prevalent plastome annotation tools. **(A)** The total citation numbers of each tools were compared, including the citation numbers of each tool per year. **(B)** The total citation numbers of all tools in different years were compared. **(C)** The change trends of citation numbers for different plastome annotation tools across different years.

The abundant citation of available plastome annotation tools (**Figure 3**) reflects the rapid increase of plastome-based studies. Although Geneious (https://www.geneious.com/) is widely used for plastome annotation, but it was not included in the comparison because it is commercial software. We compiled the total citation numbers for each tool by year, up to Sep 23, 2022, using tools from the Web of Science (**Figure 3A**). DOGMA is the most highly cited web plastome annotation tool, with 2470 citations. GeSeq has ~1200 citations, CPGAVAS has ~500 citations, and CPGAVAS2 has ~300 citations. Among command-line plastome annotation tools, PGA is the most cited, with ~500 citations. Plann has ~300 citations. The web annotation tools obviously have been more widely used than command-line plastome annotation tools. We also compiled the total citation numbers of eight tools in different years (**Figure 3B**). From 2005 to 2013, only DOGMA was available. From 2014 to 2022, more than two plastome annotation tools were published in each year. From 2005 to 2015, total yearly citation numbers of the annotation tools are gradually increased, but ≤100 times, however, from 2016 to Sep 23, 2022, the total citation numbers of the annotation tools range from ~300 to ~1000 for each year. This result indicates that the plastome-based studies rapidly increased since 2016. We also compared the changing trends of citation numbers for different plastome annotation tools across different years (**Figure 3C**). From 2005 to 2015, the citation numbers of DOGMA increased slowly, with less than 100 times, while the citation increased rapidly since 2016, and reached to top with ~500 citations in 2019. Interestingly, although the developers stated that DOGMA was no longer accepting new task submissions from 2019, it still got a lot of usages from 2019 to Sep 23, 2022 but decreased rapidly year by year (**Figure 3C**). On the contrary, citations of GeSeq, CPGAVAS and PGA increased rapidly since 2019 and achieved top citations in 2021 except 2022 with partial data from the first nine months (**Figure 3C**).

### 2.2 Common annotation errors and standardized processing

**Figure 4:**
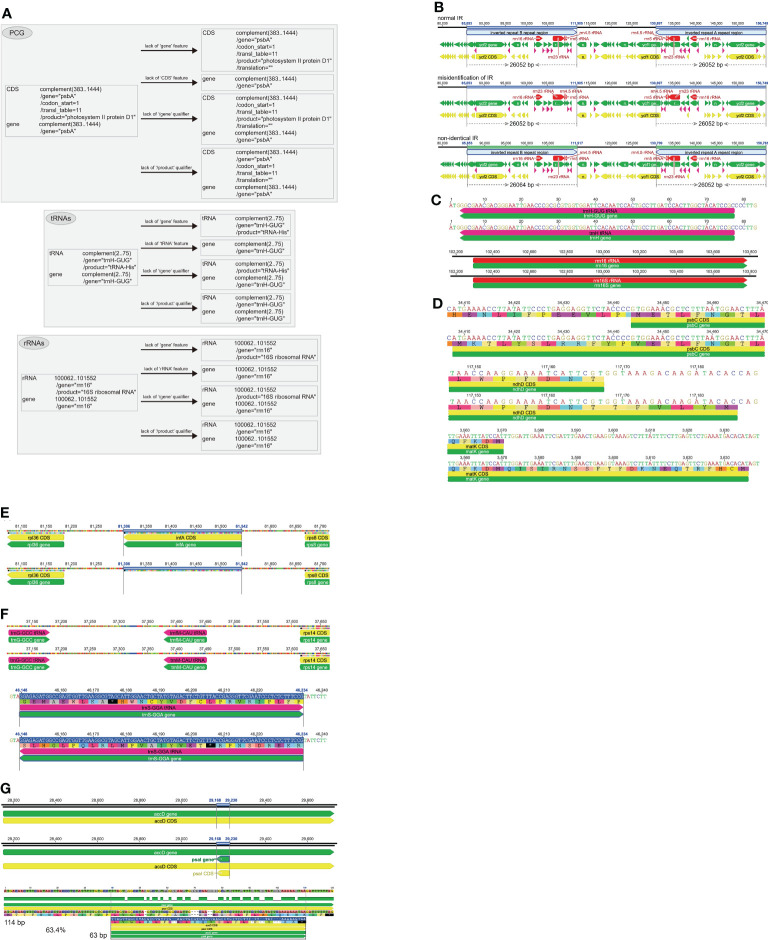
The common annotation errors in plastome. **(A)** Missing essential annotation features or qualifiers. The lack of “gene” feature, “CDS/tRNA/rRNA” feature, “/gene” qualifier, and “/product” qualifier are shown for *psbA*, *trnH-GUG* and *rrn16*, respectively. **(B)** Non-identical or misidentification of inverted repeats (IR). Three scenarios as the normal IR, misidentification of IR and non-identical IR are shown. **(C)** Non-standardized gene names. The standardized *trnH-GUG*/*rrn16* and non-standaridized *trnH*/*rrn16S* are shown in pairs. **(D)** Incorrect start codon. Three genes such as *psbC*, *ndhD* and *matK* are shown as examples. **(E)** Unannotation of known existent genes. The known existence gene *infA* between *rpl36* and *rps8* is not annotated in the plastome. **(F)** The tRNAs with wrong anticodon and incorrect coding strands. The tRNA gene *trnfM-CAU* is wrongly annotated as *trnM-CAU* with wrong anticodon. The tRNA gene *trnS-GGA* that should be annotated in the forward coding strand is incorrectly annotated in the reverse coding strand. **(G)** Gene redundancy due to annotation of nested/nonexistent genes. The gene *psaI* is shortly annotated as nested gene within *accD*. The pairwise identity value between the shortely annotated *pasI* (63 bp) nested within *accD* and the functional *psaI* (114 bp) in the reference species is as high as 63.4%, which can explain why the known nonexistent gene *psaI* is partially annotated as a nested gene within *accD*.

**Figure 5:**
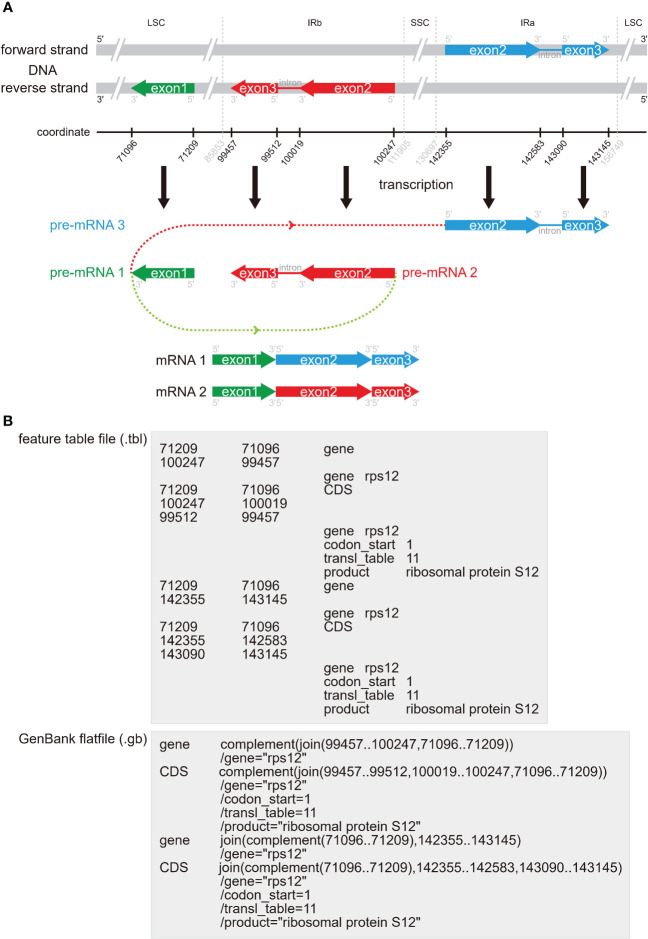
The distribution form, connect status and storage form of the trans-splicing gene *rps12* in the plastome. **(A)** The distribution of the three exons of *rps12* in the plastome and how this gene was trans-spliced. The exon 1 with green bar is in the reverse strand of the LSC region, the exon 2 and exon 3 with red bars are in the reverse strand of the IRb region, and the exon 2 and exon 3 with blue bars are in the forward strand of the IRa region. Three pre-mRNAs are generated, with the pre-mRNA 1 containing exon 1, the pre-mRNA 2 containing exon 2 and exon 3 in the IRb region, and the pre-mRNA 3 containing exon 2 and exon 3 in the IRa region. There are two mRNAs that are produced by connecting three exons of two pre-mRNAs from the same gene, with the mRNA 1 connecting the exon 1 from the pre-mRNA1 and the exon2 and exon 3 from the pre-mRNA 2, and the mRNA 2 connecting the exon 1 from the pre-mRNA1 and exon 2 and exon 3 from the pre-mRNA 3. **(B)** The correct connect status of *rps12* in the.tbl feature table file and in the.gb GenBank flatfile.

**Figure 6:**
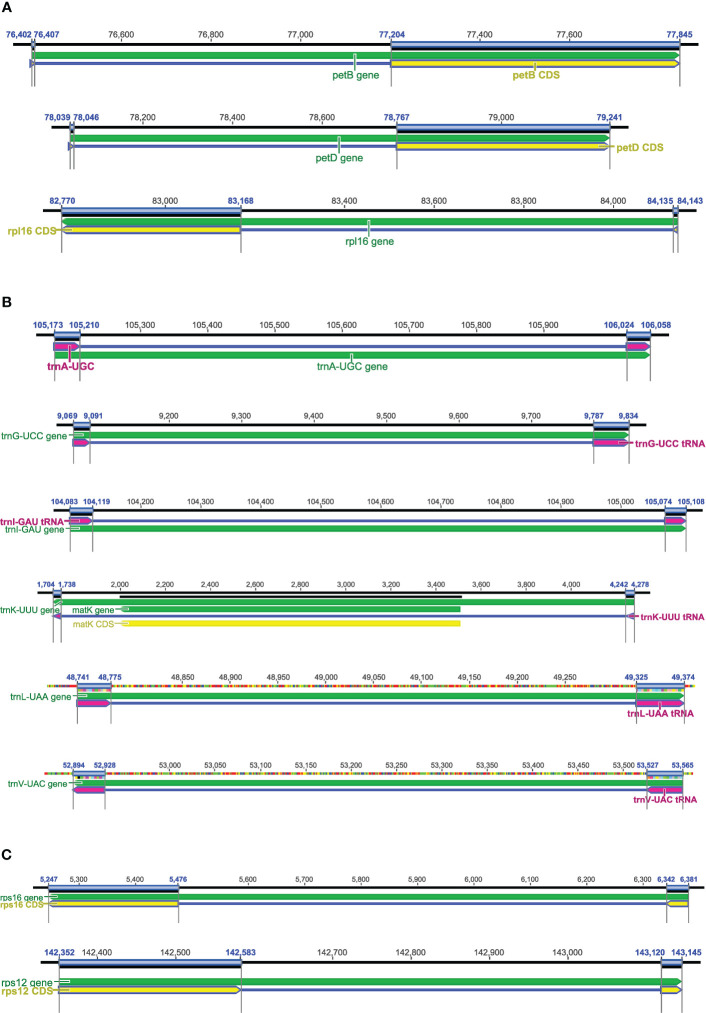
Genes with short exons in plastomes. **(A)** The short first exons in *petB*, *petD*, and *rpl16*. **(B)** The common tRNAs with introns and two short exons, e.g., *trnA-UGC*, *trnG-UCC*, *trnI-GAU*, *trnK-UUU*, *trnL-UAA*, and *trnV-UAC*. **(C)** The short exon 1 in *rps16* and short exon 3 in *rps12*.

**Figure 7:**
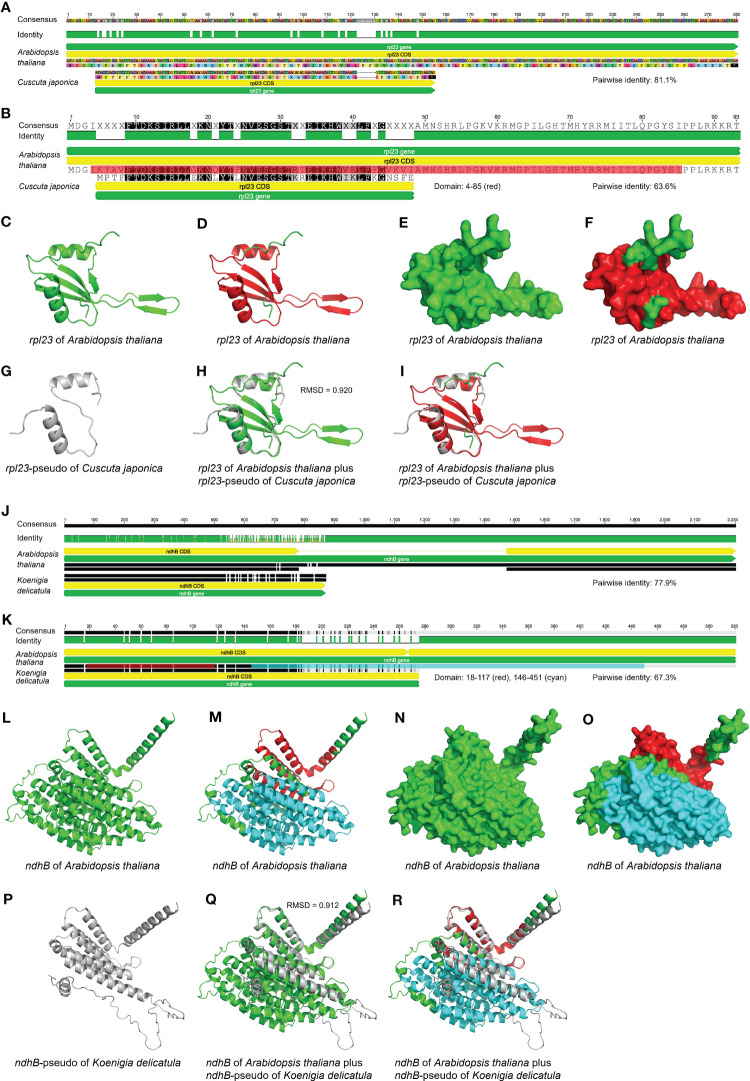
Comparison of the sequences, inferred domains and AlphaFold-predicted protein structures between potential pseudogenes and their corresponding functional genes in the plastome. **(A-I)** The sequence, domain and protein structure comparison between potential pseudogene *rpl23* in *Cuscuta japonica* and its homologous functional gene in *Arabidopsis thaliana*. **(A)** The nucleotide sequence alignment of potential pseudogene *rpl23* in *C. japonica* and its corresponding functional gene in *A. thaliana*. The pairwise identity of these two sequences is 81.1%. **(B)** The amino acid sequence alignment of potential pseudogene *rpl23* in *C. japonica* and its corresponding functional gene in *A. thaliana*. The pairwise identity of these two sequences is 63.6%. The domain of *rpl23* in *A. thaliana* shown as red color ranges from 4 to 85. **(C)** The predicted protein structure of *rpl23* in plastome of *A. thaliana* is shown in cartoon form. The protein structure is shown as green color. **(D)** The predicted protein structure of *rpl23* in plastome of *A. thaliana* is shown in cartoon form. The domain is shown as red color with the remaining residues shown as green color. **(E)** The predicted protein structure of *rpl23* in plastome of *A. thaliana* is shown in surface form. The protein structure is shown as green color. **(F)** The predicted protein structure of *rpl23* in plastome of *A. thaliana* is shown in surface form. The domain is shown as red color with the remaining residues shown as green color. **(G)** The predicted protein structure of *rpl23*-pseudo in plastome of *C. japonica* is shown in cartoon form. The protein structure is shown as grey color. Only two α-helixes of *rpl23* in *A. thaliana* are predicted for the *rpl23*-pseudo in *C. japonica*. **(H)** Comparison of the predicted protein structure of *rpl23*-pseudo in plastome of *C. japonica* with its homologous functional gene in plastome of *A. thaliana* in cartoon form. The protein structures of *rpl23* in *C. japonica* and *A. thaliana* are shown as grey and green color, respectively. The RMSD value is 0.920. The β-turns of *rpl23* in *A. thaliana* are not predicted for the *rpl23*-pseudo in *C. japonica*. **(I)** Comparison of the predicted protein structure of *rpl23*-pseudo in plastome of *C. japonica* with its homologous functional gene in plastome of *A. thaliana* in cartoon form. The protein structure of *rpl23*-pseudo in *C. japonica* is shown as grey color. The domain of *rpl23* in *A. thaliana* is shown as red color with the remaining residues shown as green color. The β-turns of *rpl23* in *A. thaliana* are not predicted for the *rpl23*-pseudo in *C. japonica*. **(J-R)** The sequence, domain and protein structure comparison between potential pseudogene *ndhB* in *Koenigia delicatula* and its homologous functional gene in *Arabidopsis thaliana*. **(J)** The nucleotide sequence alignment of potential pseudogene *ndhB* in *K. delicatula* and its corresponding functional gene in *A. thaliana*. The pairwise identity of these two sequences is 77.9%. **(K)** The amino acid sequence alignment of potential pseudogene *ndhB* in *K. delicatula* and its corresponding functional gene in *A. thaliana*. The pairwise identity of these two sequences is 67.3%. The first and second domains of *ndhB* in *A. thaliana* are shown as red and cyan color range from 18 to 117 and from 146 to 451, respectively. **(L)** The predicted protein structure of *ndhB* in plastome of *A. thaliana* is shown in cartoon form. The protein structure is shown as green color. **(M)** The predicted protein structure of *ndhB* in plastome of *A. thaliana* is shown in cartoon form. The first and second domains are shown as red and cyan color, respectively, with the remaining residues shown as green color. **(N)** The predicted protein structure of *ndhB* in plastome of *A. thaliana* is shown in surface form. The protein structure is shown as green color. **(O)** The predicted protein structure of *ndhB* in plastome of *A. thaliana* is shown in surface form. The first and second domains are shown as red and cyan color, respectively, with the remaining residues shown as green color. **(P)** The predicted protein structure of *ndhB*-pseudo in plastome of *K. delicatula* is shown in cartoon form. The protein structure is shown as grey color. Only the α-helixes in first domain of *ndhB* in *A. arabidopsis* are completely predicted for the *ndhB*-pseudo in plastome of *K. delicatula*. **(Q)** Comparison of the predicted protein structure of *ndhB*-pseudo in plastome of *K. delicatula* with its homologous functional gene in plastome of *A. thaliana* in cartoon form. The protein structures of *ndhB* in *K. delicatula* and *A. thaliana* are shown as grey and green color, respectively. The RMSD value is 0.912. Theα-helixes in second domain of *ndhB* in *A. thaliana* are not completely predicted for the *ndhB*-pseudo in *K. delicatula*. **(R)** Comparison of the predicted protein structure of *ndhB*-pseudo in plastome of *K. delicatula* with its homologous functional gene in plastome of *A. thaliana* in cartoon form. The protein structure of *ndhB*-pseudo in *K. delicatula* is shown as grey color. The first and second domains of *ndhB* in *A. thaliana* are shown as red and cyan color, respectively, with the remaining residues shown as green color. Theα-helixes in second domain of *ndhB* in *A. thaliana* are not completely predicted for the *ndhB*-pseudo in *K. delicatula*.

**Figure 8:**
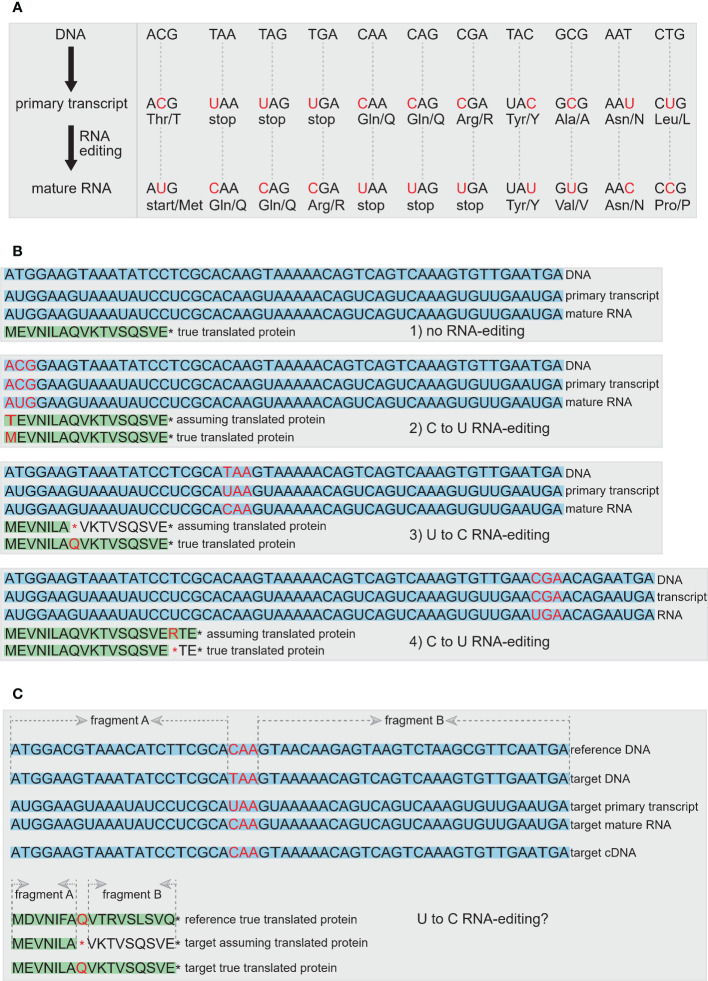
The RNA-editing events in plastomes. **(A)** RNA-editing can alter the start codon, stop codons, or result in the generation of internal stop codons, and it can also change the composition of amino acids or not. The nucleotide of the codon undergoing RNA-editing is shown in red. **(B)** Four scenarios to show the results of RNA-editing, including no RNA-editing occurred, C to U RNA-editing in generation of normal start codon in true protein compared with that in assuming protein, U to C RNA-editing in generation of longer true protein compared with assuming protein, and C to U RNA-editing in generation of shorter true protein compared with assuming protein. The DNA, primary transcript and mature RNA are shown with light-blue background. The assuming translated protein and the true translated protein are shown with light-green background. The codon, amino acid and stop codon that are associated with RNA-editing are shown in red. **(C)** A complementary method is proposed to assist in the determination of RNA-editing sites. First, if an internal/premature stop codon is occurred, the homologous sequence from the closely-related species can be used as the reference DNA to determine whether this stop codon can be restored by RNA-editing or is the signal of pseudogene. Second, we propose a quantitative standard by comparing the sequence similarity value of the aligned fragment A and fragment B between the gene from target plastome and the same one from reference plastome. If the difference value of sequence similarity between fragment A and fragment B is lower than the artificially set threshold, this gene can be preliminarily judged as an RNA-editing gene with an internal stop codon, rather than the pseudogene with a premature stop codon. Third, if the cDNA sequence from RNA-seq data is provided, the internal stop codon can be further confirmed as RNA-editing sites rather than the premature stop codon of pseudogene. In addition, the homologous protein sequence evidence from reference species, the quantitative comparison of sequence similarity value between two aligned fragments, and the true protein evidence from proteome-seq are also shown to determinate the RNA-editing sites.

Common annotation errors include, but are not limited to, missing essential annotation features or qualifiers, non-standardized gene names, non-identical or misidentification of inverted repeats (IR), incorrect start codons, failture to annotate existent genes, tRNAs with wrong anticodon and incorrect coding strands, and gene redundancy due to annotation of nested/nonexistent genes (**Figure 4**). The specific genes that need special attention are the trans-splicing gene *rps12* (**Figure 5**), genes with short exons (**Figure 6**), pseudogenes (**Figure 7**), and RNA-editing genes (**Figure 8**).

#### 2.2.1 Missing essential annotation features or qualifiers

In GenBank flatfiles, some genes lack “gene” or “CDS/tRNA/rRNA” information under FEATURE, and some genes lack “/gene” or “/product” Qualifiers (**Figure 4A**). The standardized occurrences of “FEATURES” and “Qualifiers” elements can refer to **Figure 1C**. When researchers want to write a script or program tool to parse the GenBank flatfiles, the error of “missing essential annotation features or qualifiers” must be avoided and all these essential elements must be considered (see “2.5 How to generate standardized GenBank annotation flatfiles for submission and downstream operation” for more details).

#### 2.2.2 Non-identical or misidentification of inverted repeats (IR)

The presence of IRs characterizes most plastomes, and has been considered to play an important role in stabling plastome structure through homologous recombination-induced repairs (Maréchal and Brisson, 2010), but a recent study suggests that the function of IRs remains elusive (Wang et al., 2022). The IRs are two large copies with 100% sequence identity (usually around 10 to 30 kb in length) and are readily identifiable in plastomes. However, annotation errors exist for IRs of many plastome sequences in the GenBank RefSeq database, with the two IR copies showing length differences and/or nucleotide differences (**Figure 4B**).

The short substitutions indicate sequencing errors, whereas indels and long substitutions indicate assembly errors (Turudić et al., 2022). Interestingly, all annotation tools allow for small differences between IRa and IRb, but none of them addressed the degree of differences that can be reasonably tolerated (Turudić et al., 2022). Turudić et al. (2022) proposed that further studies should be carried out to determine thresholds for the alignment parameters and which types of differences are acceptable. In fact, the sequence differences between the two IR copies may bring some mistakes to downstream analysis, such as phylogenetic reconstruction, other evolutionary analysis, or genetic engineering, because we do not know which IR copy contains the correct sequence for a target gene or intergenic region.

Although we expect the IRs to have two identical IR copies, we have to consider the impact of low quality sequences. Therefore, for standardized IR annotation we propose the following solution. We suggest running two rounds when annotating the IRs in plastome sequences. In the first run, we set the threshold of 100% percent identity for two IR copies, so the majority of plastome sequences may meet the requirement, with a minority of plastome sequences left to be further processed in the second run. However, the IRs that meet this requirement are not necessarily the true IRs, so we must ensure that all rRNAs with two gene copies are within the IR region. If this criterion is not met, the plastome sequences will be transferred to the second run. In the second run, we set the threshold value of the two IR copies to 99%, or some artificially defined threshold value that is close to 99%, so the two IR copies with some tolerated sequence differences are allowed. Sometimes it is difficult determine how many sequence differences should be tolerated. However, these cases may be very rare and can be manually checked one by one. This two-step IR annotation process is very useful in our practice.

#### 2.2.3 Non-standardized gene names

Non-standardized gene names are mainly related to tRNAs and rRNAs (**Figure 4C**). For example, *trnH-GUG* (with anticodon labeled) and *trnS* (without anticodon labeled) appear simultaneously in one plastome, or *trnH-GUG* might appear in one study and *trnH* (without anticodon labeled) in another study. These gene-name inconsistencies could lead to problems in extracting plastid genes from Genbank, and as a consequence impact downstream analysis. We strongly suggest that uniform names be adopted for plastid genes, for example, *trnH-GUG* instead of *trnH*, or *rrn16* instead of *rrn16S*. Suggested standardized gene names (and their corresponding products) for seed plant plastomes are listed in **Table 1**.

#### 2.2.4 Incorrect start codons

There are two PCGs, *psbC* and *ndhD*, whose start codons are generally not “AUG” (**Figure 4D**). For *psbC*, “GTG” has been proven to be the start codon (Kuroda et al., 2007). For *ndhD*, the RNA-editing event can alter the “ACG” to “AUG”. In some species, the genes *rps19* and *ycf1* start with “GTG”. In addition, several PCGs whose start codons could not be easily identified contain multiple “ATG” codons adjacent to the true “ATG” start codon (**Figure 4D**). In this case, it is hard to say which “ATG” is the true start codon, and we only infer that the “ATG” identical to the reference PCG based on sequence conservation is the most likely one. Therefore, the annotation quality of the reference plastome sequences have great influence on the accurate annotation of the target plastome sequences.

#### 2.2.5 Unannotation of known existent genes

Some plastome annotation tools may miss the annotation of some genes that truly exist in the plastome (**Figure 4E**). These unannotated genes may be short in length, have low sequence similarity with reference sequences, or are otherwise unrecognizabled due to the assumptions or criteria of the applied annotation tool. In this case, we recommend re-annotating these plastome sequences with annotation tools released in the last few years, such as command-line tool PGA or web tools GeSeq and Chloe, which may minimize the possibility of unannotating the existent genes. Tools as PGA and CPGAVAS2 also generate a warning file for unannotated genes for further manual check.

#### 2.2.6 tRNAs with wrong anticodon and incorrect coding strands

Several tRNAs, such as *trnS-GCU*, *trnS-UGA*, *trnS-GGA*, *trnM-CAU*, and *trnfM-CAU*, have a short length of less than 100 nucleotides and high sequence similarity with each other, which can lead to incorrect annotations (**Figure 4F**). In this case, we recommend re-annotating these plastome sequences with the recently released annotation tools noted above (PGA, GeSeq, CPGAVAS2, and Chloe), most of which have developed specific algorithms or integrated third-party tools to minimize the possibility of annotation of wrong tRNAs or annotation in incorrect coding strands.

#### 2.2.7 Gene redundancy due to annotation of nested/nonexistent genes

There are some genes that can be annotated as nested genes, and/or some short nonexistent gene fragments can be annotated accidentally (**Figure 4G**). Both of these two cases can lead to gene redundancy. This is mainly caused by the wrong annotation of fragments in the target plastomes that are highly similar to fragments of their reference genes. For these redundantly annotated genes, we suggest recording them in the log file. For other annotation tools which do not consider gene redundancy, additional functionality could be developed that imposes constraints with regard to nested genes and gene length. In any case, it is always difficult to distinguish pseudogenes from redundant genes, especially in highly divergent plastomes (see “2.2.10 Pseudogenes” for more detailed discussions). To a certain extent, annotation of nested genes is easier to avoid than annotation of nonexistent genes.

#### 2.2.8 Trans-splicing gene *rps12*

Trans-splicing refers to the linking of two or more exons from two different pre-mRNAs. In contrast to canonical cis-splicing, the two or more exons are from different pre-mRNAs, but also may be from the same gene. The *rps12* gene is a trans-splicing gene and is often incorrectly or incompletely annotated. The *rps12* contains three exons of two pre-mRNAs, including exon 1 in the LSC region and exon 2 and exon 3 in the IR regions, respectively (**Figure 5**). Even if we manually annotate *rps12*, it is usually difficult to correctly connect these three exons. As far as we know, CPGAVAS2 and GeSeq tried to automatically connect the exons of *rps12*, while quantitative assessment of their accuracy has not been conducted. In **Figure 5**, we show the distribution of the three exons of *rps12* in plastome (**Figure 5A**), how this gene is trans-spliced (**Figure 5A**), and how this gene is displayed in the.tbl feature table files and in the.gb GenBank flatfiles (**Figure 5B**). Based on the annotation standard of *rps12*, you can easily manually modify the.tbl feature table files and the.gb GenBank flatfiles to correct the connecting status. When adjusting the connecting status of *rps12*, if you have only modified the.tbl feature table and do not want to edit the.gb GenBank flatfile, you can use the table2asn tool to automatically transform the.tbl file to the.gb file.

#### 2.2.9 Genes with short exons

The plastid genes *petB*, *petD*, and *rpl16* contain short exons, and each of these genes includes a short first exon and a much longer second exon (**Figure 6A**). The short first exon is usually hard to be detected using current BLAST search methods, while the second exon is long enough to be located. As far as we know, DOGMA, CPGAVAS and GeSeq have not designed additional algorithms to accurately identify and annotate the short first exons, while CPGAVAS2, PGA, and Chloe have developed relevant algorithms for the accurate annotation of these genes. For the first short exons of these three genes, the performance of these tools needs to be tested. For those tRNAs with introns (e.g., *trnA-UGC*, *trnG-UCC*, *trnI-GAU*, *trnK-UUU*, *trnL-UAA*, *trnV-UAC*), the tRNA exon lengths are also short (**Figure 6B**). In addition, exon 1 of *rps16* and exon 3 of *rps12* are also short, but much longer than exon 1 of *petB*, *petD*, and *rpl16* (**Figure 6C**). In some angiosperm lineages, the gene *rps16* is relatively divergent. So we also suggest testing the performance of the above mentioned tools for annotation of these tRNAs, *rps16*, and *rps12*, particularly regarding whether or not they are annotated, whether or not all exons are recognized, and the accuracy of exon-intron boundaries. It is known that RNA-seq and/or proteome-seq data is important for the detection of short exons in PCGs, but not for those in tRNAs.

#### 2.2.10 Pseudogenes

Pseudogenes are non-functional gene fragments that are derived from and similar to functional genes. Any genes may become pseudogenes due to mutations resulting in premature stop codons or frameshits. Although pseudogenes have partial fragments similar to known functional genes, they are usually degraded due to the general lack of selective constraints. The existence of pseudogenes complicates genome annotation, and it is often difficult to determine whether a gene is functional or non-functional. As far as we know, there are few systematic studies on plastid pseudogenes. In heterotrophic plants such as parasitic and carnivorous plants, pseudogenes frequently appear in their plastomes, while they are rare in the plastomes of autotrophic plants.

Pseudogenes and functional genes are not easy to distinguish due to the existence of RNA-editing events. Although premature stop codons are frequently used as a criterion for recognizing pseudogenes, RNA-editing, although rare in seed plants, can convert premature stop codons to non-stop codons (see “2.2.11 RNA-editing genes” and **Figure 8** for more details). Therefore, we should carefully judge whether genes with premature stop codons are indeed genuine pseudogenes or instead functional genes with RNA-editing sites. However, it is hard to determine this without RNA-seq data.

Plastome annotation tools generally annotate pseudogenes as normal genes, and let the user to judge whether these genes are functional or pseudogenized, but this can result in incorrect pseudogene identification as well. For example, *matK* is an essential splicing factor and was previously described as a pseudogene in many photosynthetic orchid species due to the presence of a premature stop codon caused by a frameshift mutation. A study by Barthet et al. (2015) identified an out-of-frame alternative “AUG” initiation codon upstream from the common initiation codon used for translation of *matK* in other angiosperms. The alternative translation initiation codon can generate a conserved reading frame encoding functional MatK protein (Barthet et al., 2015).

Several source of evidence are needed to determine the existence of pseudogenes. First, we recommend using closely-related species as the reference for annotation. The reference plastomes need to be carefully checked, and the target plastomes annotated based on the reference plastomes also need to be carefully checked. If there is an annotation error for a gene in the reference plastome, this error will be spread to the annotated plastomes. Second, RNA-editing events that can cause the misidentification of genuine functional genes as non-functional pseudogenes should be considered. Third, gene length comparisons between target genes and reference genes can be useful. PGA has tried to detect putative pseudogenes based on a built-in parameter defined by dividing the length of the annotated gene by that of the reference gene (Qu et al., 2019). However, this method may not be reliable for plastomes with abundant RNA-editing sites. For gene-length difference comparisons, the sorted gene names in descending order of gene length difference can refer to **Table 3** (see “2.4.2 Gene length difference comparison”). Fourth, sequence similarity comparison is a good quantitative method; we suggest considering not only the sequence similarity for the regions between the start and premature stop codons in the target gene, but also the sequence similarity for the regions between the premature stop codon in the target gene and the stop codon in the reference gene. If the difference in sequence similarity between these two alignment fragments does not exceed a set threshold, the genes may be functional genes with sequencing or assembly errors. Fifth, RNA-seq data is important for judging the expression status of the genes. Six, the identification of domains is also helpful to determine whether a gene is pseudogene or not, due to the functional nature of domains. If a domain exists in the gene, functional protein may be translated. The Pfam database integrated in InterPro of UniProt (https://www.uniprot.org/) is a useful resource that can classify protein sequences into families and predict the presence of domains. Seven, the final and decisive operation is to acquire the amino acid sequences from proteome-seq data. Protein structure prediction seems to be the optimal alternative operation to proteome, which is easy to achieve thanks to currently available tools, such as AlphaFold (Jumper et al., 2021; https://github.com/deepmind/alphafold) and AlphaFold Protein Structure Database (Varadi et al., 2022; https://www.alphafold.ebi.ac.uk/). There is an online AlphaFold for predicting protein structures (https://colab.research.google.com/github/sokrypton/ColabFold/blob/main/AlphaFold2.ipynb). SWISS-MODEL (https://swissmodel.expasy.org/) is another popular online tool for the prediction of protein structures. PyMOL (https://pymol.org/2/) is an excellent tool for performing protein structure comparison and visualization. Although only parts of proteins from specific species are present in the Protein Data Bank (PDB) database and many protein structures are determined by only a fragment of the sequence, the experimental protein structures deposited in the PDB are still important references for comparing plastid protein structures. For PCGs in plastomes, protein structure predictions and comparisons have been seldom used in previous studies.

**Table 3:** Example comparison of gene length differences for assessing plastome annotation quality.

	gene name	target lenght	reference lenght	length difference
PCGs	*ycf1*	600	5000	1000
	*psbA*	1200	1000	200
	*rbcL*	1500	1500	0
	*ycf2*	4000	5000	-1000
	*ndhA*	2000	2100	-100
	*matK*	1000	1010	-10
tRNAs	*trnS-GCU*	75	72	3
	*trnI-CAU*	72	72	0
	*trnH-GUG*	70	72	-2
	*trnS-GGA*	71	72	-1
rRNAs	*rrn16*	2010	2000	10
	*rrn23*	3000	3000	0
	*rrn5*	980	1000	-20
	*rrn4.5*	490	500	-10

For comparison of domains and protein structures, we took pseudogene *rpl23* in *Cuscuta japonica* and pseudogene *ndhB* in *Koenigia delicatula* as examples to show their structural difference compared to corresponding functional genes in *Arabidopsis thaliana*, and try to explain why these two genes are pseudogenes from the view of domains and protein structures (**Figure 7**). In this review, we also showed the inferred functional domains (**Figure S1**; **Table S1**) and AlphaFold-predicted protein structures (**Figure S2**) for all PCGs occurred in seed plants, for *infA* in *Amborella trichopoda*, for *chlB*/*L*/*N* and *psaM* in *Zamia furfuracea*, and for remaining PCGs in *Arabidopsis thaliana*. This should help as well for determining whether a gene is in fact a pseudogene.

#### 2.2.11 RNA-editing genes

RNA-editing is a post-transcriptional modification of RNA that occurs in nuclear and organellar genomes (Maier et al., 1996; Gott and Emeson, 2000). Modifications due to RNA-editing involve nucleotide substitutions and insertions or deletions that can affect both protein-coding and non-protein coding RNAs (Maier et al., 1996). After the discovery of cytidine-to-uridine (C-to-U) RNA-editing in plant mitochondrial genomes (Covello and Gray, 1989; Gualberto et al., 1989; Hiesel et al., 1989), and soon thereafter in plastomes (Hoch et al., 1991), RNA-editing in organellar genomes has gained more and more attention (Lenz et al., 2018), especially due to the extensive availability of plant organellar genomes. In plants, RNA-editing occurs mostly in organelles in the form of C-to-U conversion, albeit the opposite U-to-C event has been observed in some taxa, especially chloroplast RNAs (Takenaka et al., 2013). Most RNA-editing events in protein-coding regions tends to modify affected codons by restoring conserved amino acid residues or to create start and stop codons (Chateigner-Boutin and Small, 2011; Takenaka et al., 2013). An apparent absence of RNA-editing from the plastome has been found in *Equisetum hyemale* and *Welwitschia mirabilis* (Guo et al., 2015; Fan et al., 2019). In plastomes of some plant groups such as hornworts and ferns, RNA-editing can occur in up to 78% of protein-coding genes (Kugita et al., 2003; Wolf et al., 2004).

RNA-editing events can often hinder the accurate annotation of functional genes in plastomes. Many RNA-editing sites will alter the sequences of start codons, stop codons, or result in internal stop codons within the coding sequences of plastomes (**Figure 8**). Most currently available plastome annotation tools do not consider the annotation of RNA-editing sites, which can lead to some annotated genes without normal start codons or stop codons or with some internal stop codons. Consequently, these issues result in some plastid sequences that do not meet the requirements for submission to public databases such as GenBank (Robison and Wolf, 2019). Specifically, some plastid genes with abnormal start codons or stop codons or internal stop codons are not easily verified as genes experienced RNA-editing event vs. pseudogenes. The existence of RNA-editing sites in coding regions of plastomes may have more or less effect on phylogenetic reconstruction (Bowe and DePamphilis, 1996; Du et al., 2020). Therefore, the impact of RNA-editing on gene prediction needs to be considered during the annotation process, particularly for the clades with hundreds or thousands of RNA-editing sites, such as hornworts, lycopods, and ferns (Takenaka et al., 2013). Labeling RNA-editing sites may be a requirement for standardizing plastome annotation, which needs to be added to current plastome annotation tools. There are some independent tools that can predict and annotate RNA-editing sites in plastomes, such as PREP suite (Mower, 2009), ChloroSeq (Smith and Sanitá Lima, 2017), PREPACT 3.0 (Lenz et al., 2018), and ReFernment (Robison and Wolf, 2019), and there are databases collecting plant organellar RNA-editing events and visualizations of amino acid changes, such as REDIdb 3.0 (Lo Giudice et al., 2018). For example, ReFernment runs under the assumption that only U-to-C or C-to-U RNA-editing appears in the plastome (Takenaka et al., 2013), and all nonsense mutations are the result of RNA-editing. So if a gene has incorrect start or stop codons, is frameshifted, or the generated sequence has other sequencing or assembly errors, ReFernment might recognize these cases as RNA-editing rather than other errors (Robison and Wolf, 2019).

Previous studies showed that the RNA-editing sites can be verified by comparing genomic sequences with cDNA sequences (**Figure 8C**; Wolf et al., 2004), or can be predicted by comparing genomic sequences with verified DNA or amino acid sequences among closely-related species (**Figure 8C**; Lenz et al., 2018). If there are more than a certain number of internal stop codons in a gene, we suggest outputing an error warning file so the user can conduct a manual check. Plastid genes with more than five internal stop codons caused by RNA-editing are relatively rare (Robison and Wolf, 2019), and so manual checking should be the most efficient way to resolve these issues. In **Figure 8C**, we propose a complementary method to assist in the determination of RNA-editing sites.

### 2.3 Establishing database of reference plastomes with standardized annotations

Although many plastome sequences have been assembled and published, it remains difficult to determine which are best for use as reference plastomes for annotation. In light of this, the community would benefit from the establishment of a database of reference plastome sequences with standardized annotations for each family (or even for each genus), and the database could be periodically updated. In order to facilitate citation and reuse, we also suggest creating unique accession number for these reference plastomes. We suggest that when annotating the target plastomes, it is necessary to use the plastomes of the species with the highest priority as the reference plastomes, which is similar to the type specimen in species identification. A recently published study generated plastome database with curated plastomes (Hua et al., 2022). It is a good to help researchers to resolve various plastome annotation problems.

### 2.4 Quantitative judgement standards of high-quality plastome annotation

Based on the common annotation errors and potential solutions described above, we provide a set of standards for evaluating plastome annotations for the scientific community. These are outlined below.

#### 2.4.1 Gene number comparison

Benchmarking Universal Single-Copy Orthologs (BUSCO), using conserved core orthologous genes as reference to judge the annotation status of genes in newly sequenced genomes, is designed for assessing the completeness of nuclear genome assembly (Simão et al., 2015). This method could also be used for assessments of plastome annotations. Considering the limited gene number in plastomes, we suggest simplifying the BUSCO assessment parameter and propose a standard for assessing annotation completeness of genes as nHMG (number of hitting and missing genes). There are five numbers that would be calculated, i.e., number of hitting genes (nH), number of missing genes (nM), percent of hitting gene numbers (pH), percent of missing gene numbers (pM), and total gene numbers (n); additionally the following six numbers should be calculated for PCGs, tRNAs, and rRNAs, respectively, i.e., number of hitting PCGs (nHP), number of missing PCGs (nMP), number of hitting tRNAs (nHT), number of missing tRNAs (nMT), number of hitting rRNAs (nHR), and number of missing rRNAs (nMR). Since plastomes do not contain many genes (~100), it would be reasonable for users to manually check the status (hitting vs. missing) for each gene. Users then can determine why genes are absent (true loss vs. missing annotation for a gene that is actually present). An example of this type of gene number comparison (nHMG) is shown in **Table 2**.

#### 2.4.2 Gene length difference comparison

Gene lengths of PCGs, tRNAs and rRNAs are highly conserved across plant species, with rare exceptions. Therefore, annotation accuracy of plastomes can be further assessed by comparing length differences between genes in the target plastome and their corresponding genes in the reference plastome. Gene differences can then be sorted by magnitude, and those with the largest differences (or those larger than a specified threshold) can be checked. In this case, we suggest comparing the length differences of PCGs, tRNAs, and rRNAs separately. An example is shown in **Table 3**.

#### 2.4.3 Gene sequence similarity comparison

Because plastid gene sequences, especially those of tRNAs and rRNAs, are highly conserved, sequence similarity between target and reference genes can be compared and sorted (by percent similarity) to identify genes above/below a specified threshold for manual checking. As with the other examinations, we suggest comparing PCGs, tRNAs, and rRNAs separately. Current plastome annotation tools consider the sequence similarity of homologous genes, but are generally not explicit or flexible regarding thresholds of sequence similarity for gene annotation. It is also generally worthwhile to manually examine alignment files with regard to sequence conservation. Finally, users can comprehensively judge whether the annotated genes are redundant according to the sequence similarity values and the alignment files. An example for comparisons of gene sequence similarity is shown in **Table 4**.

**Table 4:** Example comparison of gene sequence similarity for assessing plastome annotation quality.

	gene name	alignment lenght	sequence similarity
*PCGs*	*ycf1*	6000	41.24%
	*psbA*	1200	53.48%
	*rbcL*	1500	80.32%
	*Ycf2*	4000	98.81%
	*ndhA*	2000	99.99%
	*matK*	1000	100%
*tRNAs*	*trnS-GCU*	75	89.20%
	*trnI-CAU*	72	94.00%
	*trnH-GUG*	70	99.75%
	*trnS-GGA*	71	100%
*rRNAs*	*rrn16*	2010	94.63%
	*rrn23*	3000	96.44%
	*rrn5*	980	97.93%
	*rrn4.5*	490	100%

### 2.5 How to generate standardized GenBank annotation flatfiles for submission and downstream analysis

The storage form of annotation information is important for plastome annotation. The GenBank flatfile is a common template file for storing annotation information of genome sequences, and it can be easily recognized and manipulated using a variety of softwares. If the GenBank flatfile for plastome annotations is standardized, operations such as submission to GenBank and extraction of genes and relevant sequences will become much easier. However, errors are often present in GenBank flatfiles (Smith, 2012), even when GenBank flatfiles following the RefSeq standards are downloaded directly from GenBank. Therefore, we provide instructions on generating standardized GenBank flatfiles for submission and downstream analysis.

First, plastome sequences should be submitted to GenBank. Depositing annotated sequences to GenBank is an essential step before publication of research results, but this operation is generally inefficient, time-consuming, and tedious (Smith, 2020). Submission instructions and submission preparation tools (https://www.ncbi.nlm.nih.gov/genbank/submit/) are well documented by NCBI, which can assist with the submission of any type of sequence. For submission of plastome sequences with annotation information to GenBank, we suggest to using Banklt (https://www.ncbi.nlm.nih.gov/WebSub/), a web-based submission tool allowing automatic submission to GenBank. It is easy to fill out the submission template information including locus, definition, accession, organism, reference authors, reference title, and reference journal, all found in the head annotation section of the GenBank flatfiles (.gb/.gbf/.gbk). The GenBank flatfiles can be downloaded from GenBank, but these files are not allowed to be directly submitted to GenBank. So two files need to be prepared separately, including the sequence file in FASTA format (.fsa/.fasta/.fa/.fas) and the 5-column feature table file (.tbl). These two files can be generated by transforming the GenBank flatfiles using the gbf2tbl.pl Perl script provided by NCBI. As the input file of gbf2tbl tool, the incomplete GenBank flatfiles without the head annotation section (e.g., LOCUS, DEFINITION, ACCESSION, and ORGANISM.) are allowed, because this information is not required to generate the.fsa and the.tbl files. Furthermore, when using Banklt, you can generate the head annotation section of the GenBank flatfiles by filling in the corresponding location. That is to say, if you just want to submit the plastome sequences to GenBank, the GenBank flatfiles generated by plastome annotation tools can be incomplete and only retain the annotation and sequence content below the line “FEATURES Location/Qualifiers”. It is also fine if the start-line beginning with the word “LOCUS” is retained.

Second, GenBank flatfiles should be generated for downstream analysis. The head annotation section in the GenBank flatfiles is not necessary when you submit plastome sequences to GenBank, but this content provide some basic statistics useful for extracting information from GenBank flatfiles. The standard GenBank flatfiles of plastome sequences downloaded from GenBank include the head annotation section, which is generated by GenBank based on the information supplied during the Banklt submission process. Currently, some plastome annotation tools like GeSeq and CPGAVAS2 can generate complete GenBank flatfiles and allow users to modify the information included in the head annotation section. However, it is not easy to accurately modify the GenBank flatfiles for those who are not bioinformatics experts. Therefore, it is best to automatically generate head annotation sections that do not require major modifications. This online tool (https://submit.ncbi.nlm.nih.gov/genbank/template/submission/) can be used to prepare the submission template by yourself, and then the generated submission template file (.sbt) can be downloaded to personal computers. Standard GenBank flatfiles can then be generated by adding the content in the.sbt submission template file. When using table2asn (https://www.ncbi.nlm.nih.gov/genbank/table2asn/; table2asn is the replacement of the older now-obsolete tool tbl2asn), three types of files, including the.sbt submission template file, the.tbl feature table file, and the.fsa sequence file, are required to generate standard GenBank flatfiles. It should be noted that, after this transformation, amino acid sequences of each PCG that may not have existed in the initial GenBank flatfiles will then be present following the qualifier “/translation”.

As mentioned above, we all know that the.gb GenBank flatfile and the.tbl feature table file are important for the display of plastome annotation information. If the.gb file contains errors, the.tbl file will also contain errors, and vice versa. Thus we propose to development of scripts or tools to check whether these two important files are complete and following established standards, and whether some essential annotation information in these files is incorrect or even missing.

## 3 Prospect

### 3.1 Integrated plastome annotation approaches

In recent years, some plastome annotation tools have been developed to facilitate rapid annotation of sequenced plastomes, and these apply different approaches or criteria for gene prediction and correction. The types of information used for annotation include, but are not limited to, homology-based gene prediction, RNA-seq read mapping, domains, and proteome-seq amino acid sequence alignment. In the future we hope to see an integrated plastome annotation tool that combines diverse sources of information to achieve comprehensive and accurate annotation. Most genes will be annotated indentically using different sources of information, but those genes that show different annotation results based on different sources of information can be manually checked and curated using tools such as Geneious (https://www.geneious.com/). In addition, a quantitative approach is also important for assessing the annotation quality of plastomes. In this review, we have proposed several assessment standards that can help user to evaluate plastome annotation quality, such as gene number comparisons, gene length difference comparisons, and gene sequence similarity comparisons. Other quantitative approaches could be developed as well, such as the percentage of genes substantiated by RNA-seq or proteome-seq technologies.

### 3.2 Annotation of other features on plastomes

Most plastome annotations have focused on coding genes and ignored or paid insufficient attention to other intriguing features, e.g., sRNAs, promoters, UTRs, transcription initiation sites, and ribosome binding sites. At the present, none of the currently available plastome annotation tools can identify these features. In the study of Ruwe and Schmitz-Linneweber (2012), they mined the sRNAs and explored their potential regulatory roles within chloroplasts. Future plastome annotation tools should attempt to integrate new functionalities to examine these other features of plastomes.

## Author contributions

X-JQ and T-SY designed the outline of this review. X-JQ, DZ, and R-YZ collected and analyzed the data with inputs from GS. X-JQ wrote the first draft of the manuscript. T-SY and GS revised the manuscript with inputs from all authors. All authors contributed to the article and approved the submitted version.

## Supplementary material

Supplementary Figure 1 The inferred functional domains for all PCGs occurred in seed plants, for *infA* in *Amborella trichopoda*, for *chlB*/*L*/*N* and *psaM* in *Zamia furfuracea*, and for remaining PCGs in *Arabidopsis thaliana*. The domain names and their coordinates are shown, with 1^st^ to 79^th^ PCGs sharing a coordinate and with 80^th^ to 83^th^ PCGs sharing another coordinate.

Supplementary Figure 2 The AlphaFold-predicted protein structures for all PCGs occurred in seed plants, for *infA* in *Amborella trichopoda*, for *chlB*/*L*/*N* and *psaM* in *Zamia furfuracea*, and for remaining PCGs in *Arabidopsis thaliana*. The protein structures are sorted according to the gene names in **Table S1**. The first column indicates the predicted protein structure in cartoon form, with the protein structure shown as green color. The second column indicates the predicted protein structure in cartoon form, with the domain shown as red color and the remaining residues shown as green color. The third column indicates the predicted protein structure in surface form, with the protein structure shown as green color. The fourth column indicates the predicted protein structure in surface form, with the domain shown as red color and the remaining residues shown as green color.

Supplementary Table 1 The inferred functional domains for all PCGs occurred in seed plants, for *infA* in *Amborella trichopoda*, for *chlB*/*L*/*N* and *psaM* in *Zamia furfuracea*, and for remaining PCGs in *Arabidopsis thaliana*.
