# Supplementary figures and images for: Progress, challenge and prospect of plant plastome annotation
Source: Front Plant Sci. 2023 May 30;14:1166140. doi: 10.3389/fpls.2023.1166140 (PMC10266425; doi:10.3389/fpls.2023.1166140)

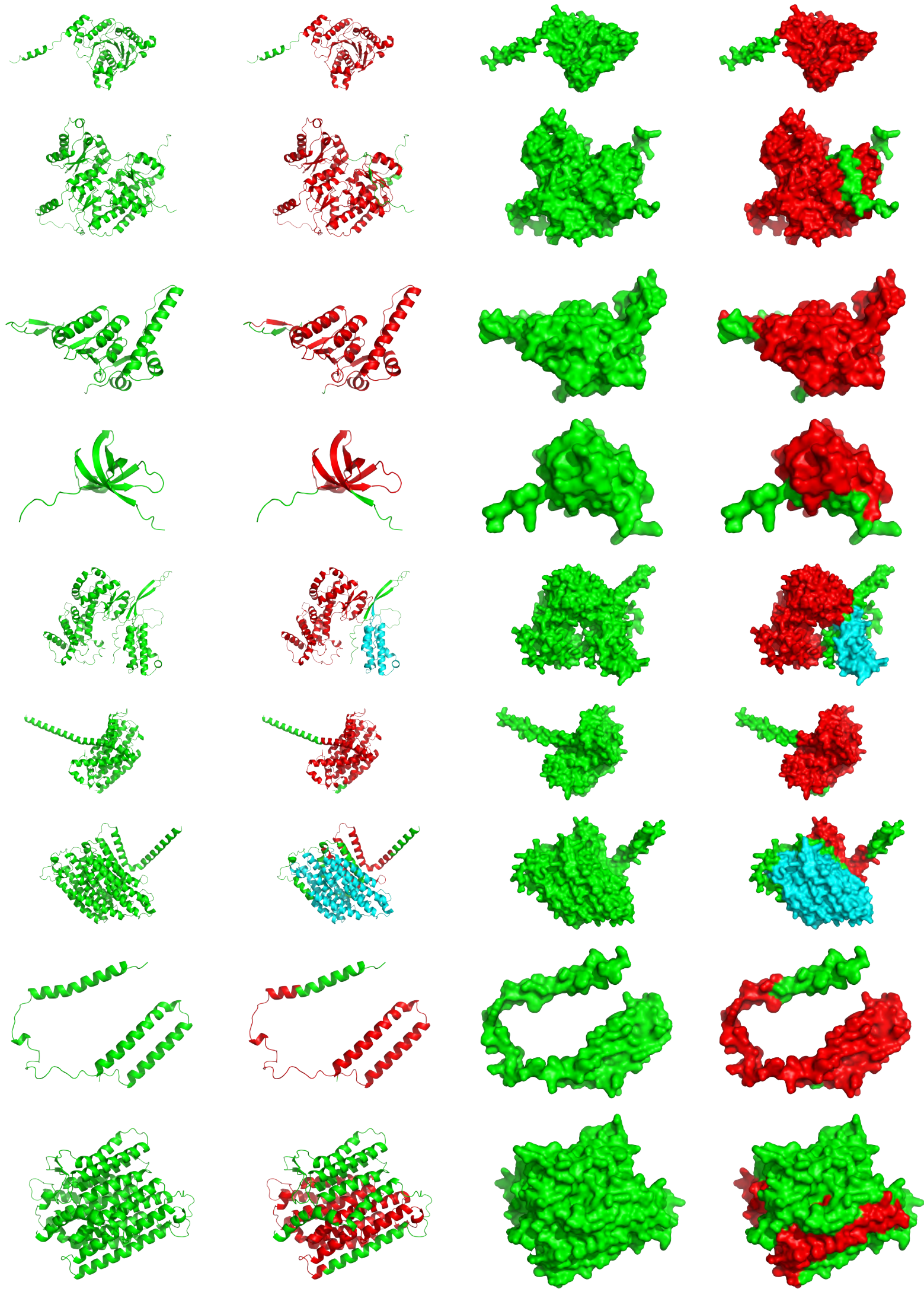

Supplement: Supplementary file 1 [file DataSheet_3.pdf]

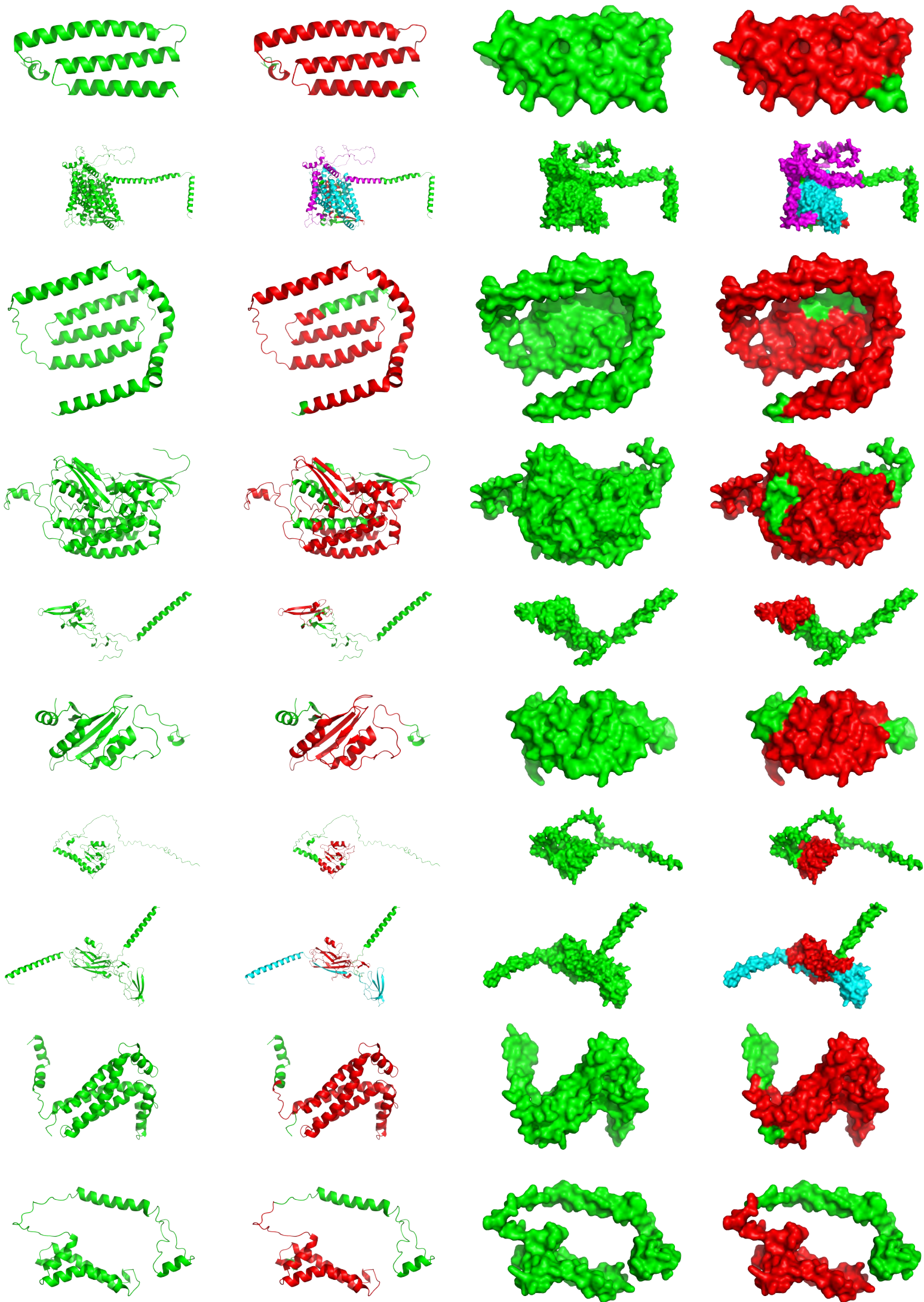

Supplement: Supplementary file 2 [file DataSheet_4.pdf]

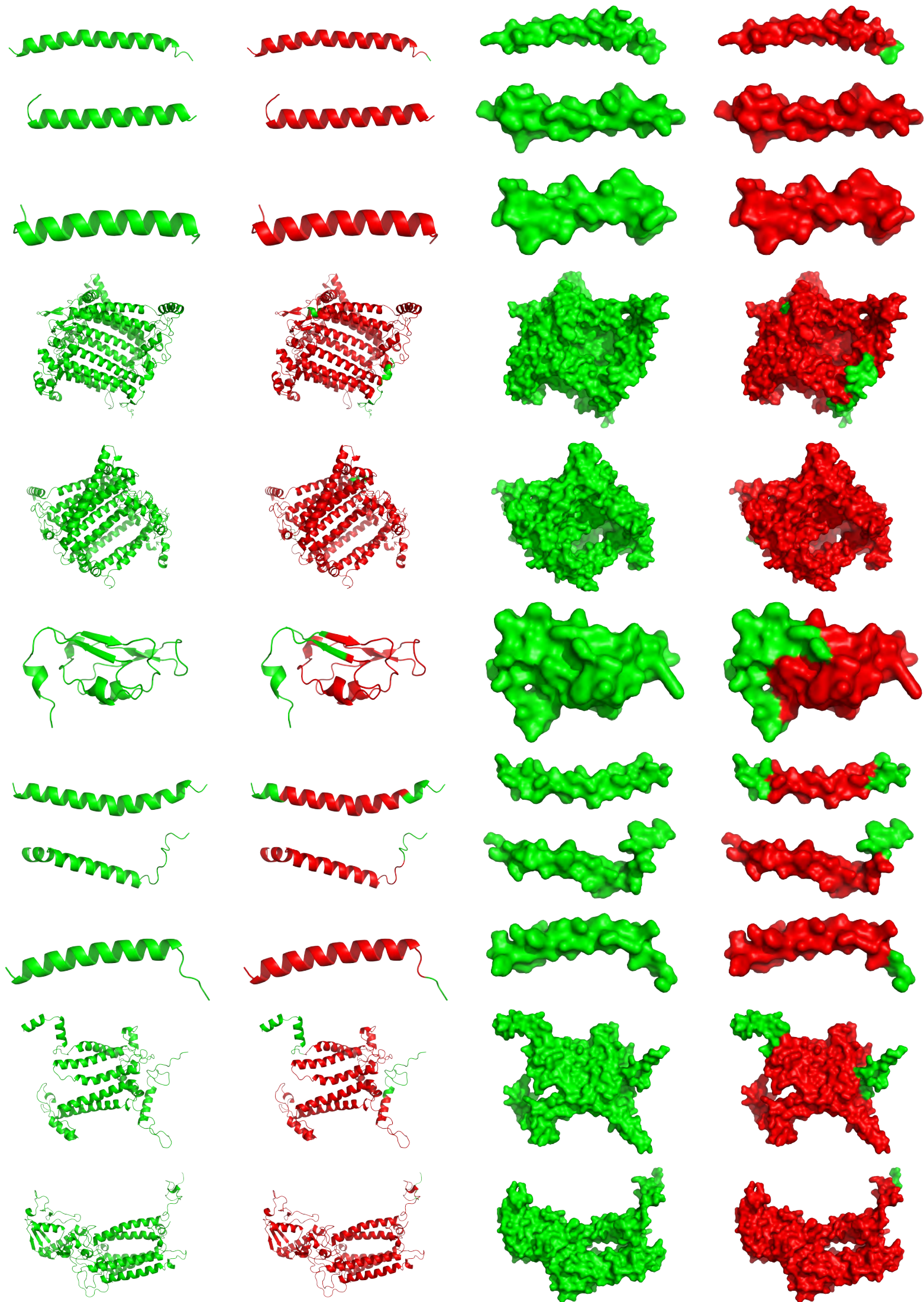

Supplement: Supplementary file 3 [file DataSheet_5.pdf]

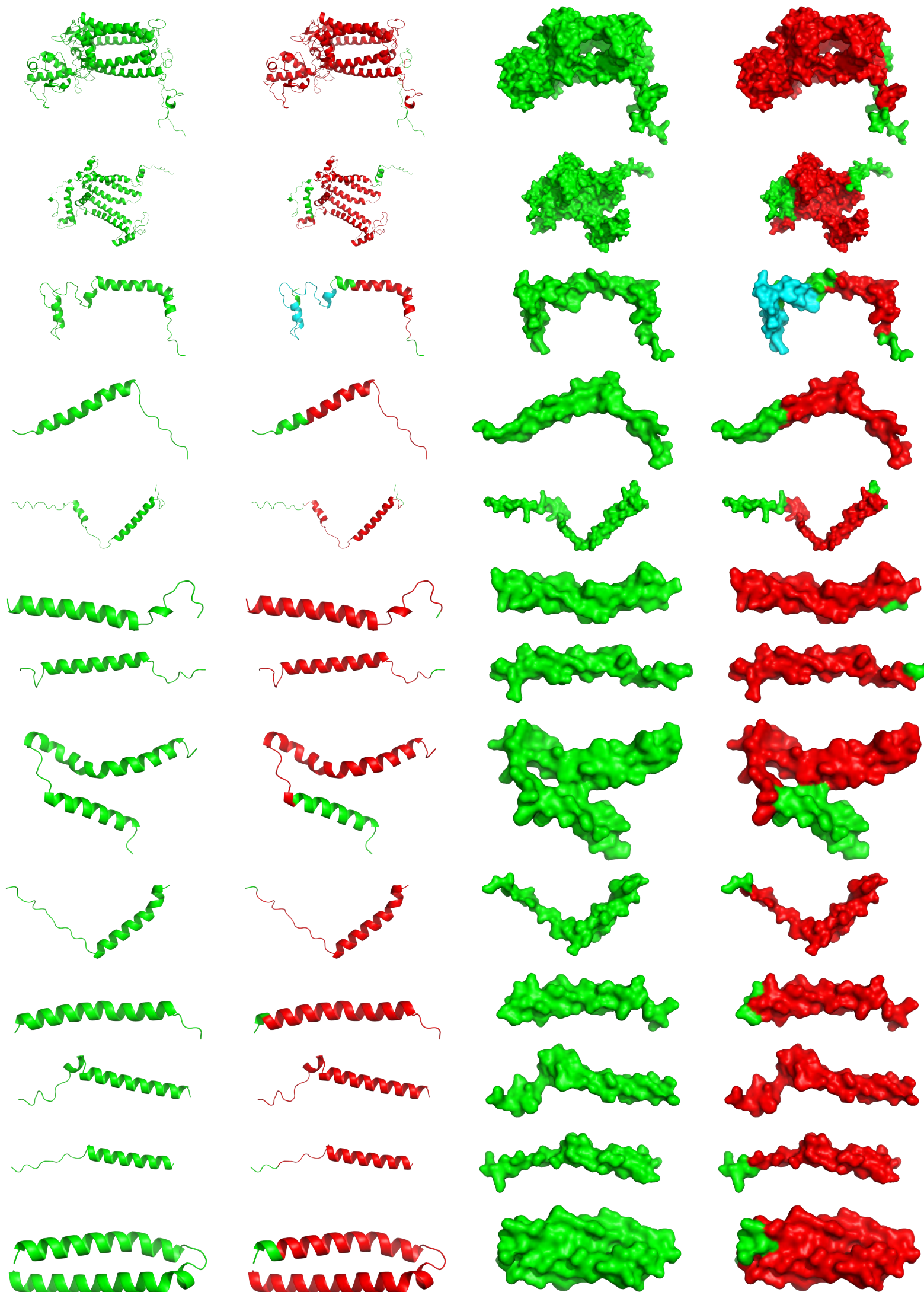

Supplement: Supplementary file 4 [file DataSheet_6.pdf]

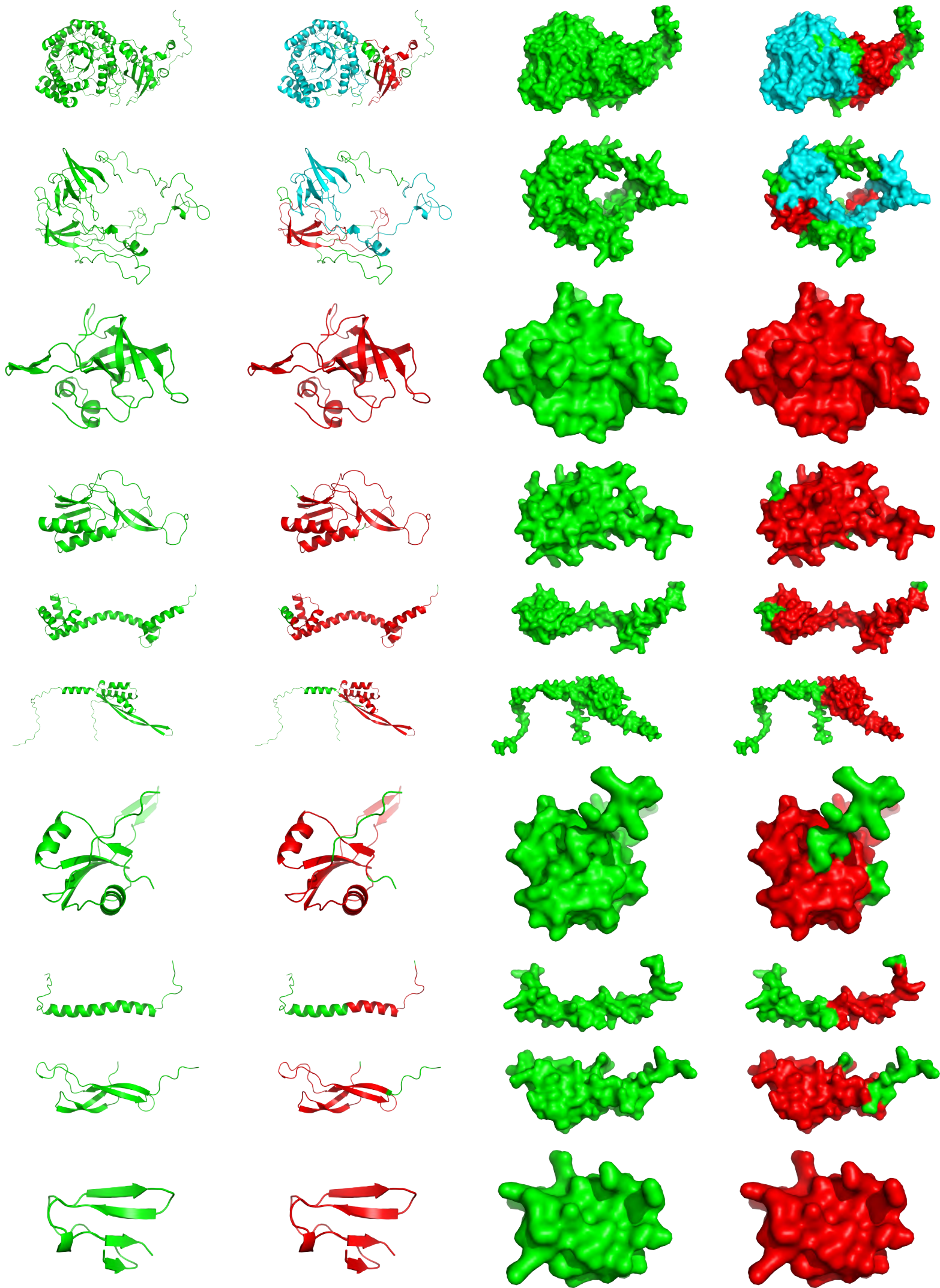

Supplement: Supplementary file 5 [file DataSheet_7.pdf]

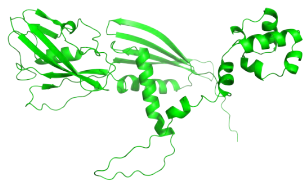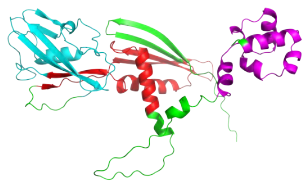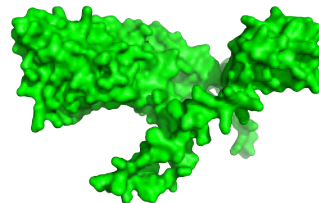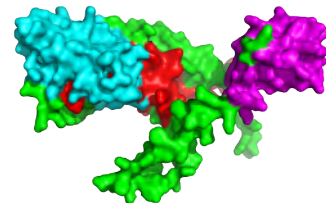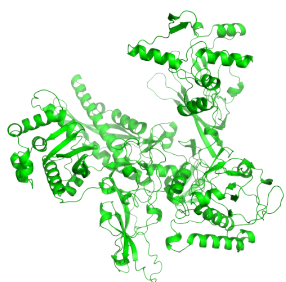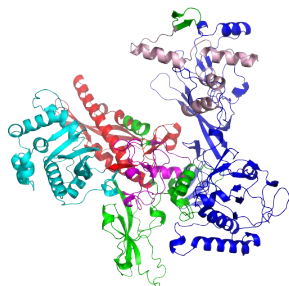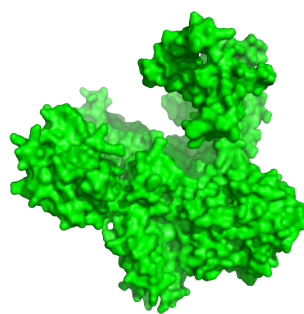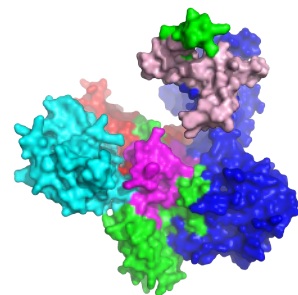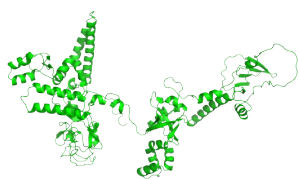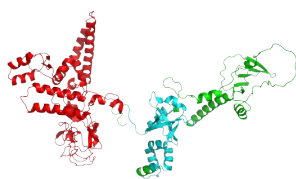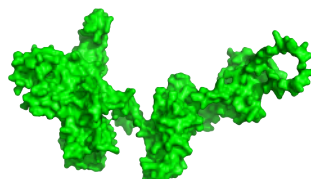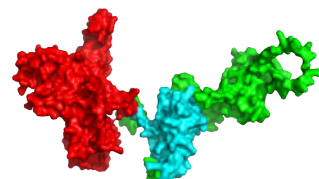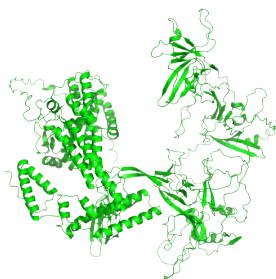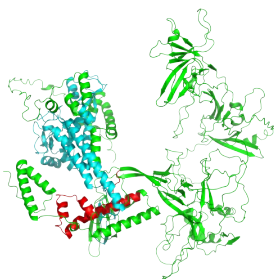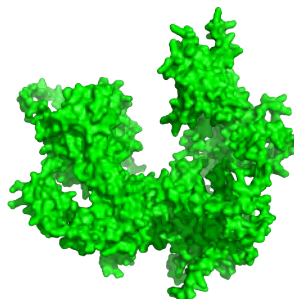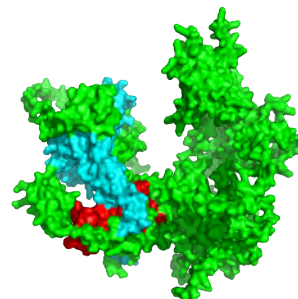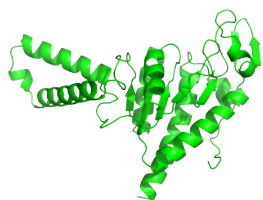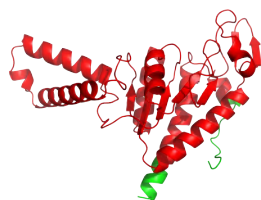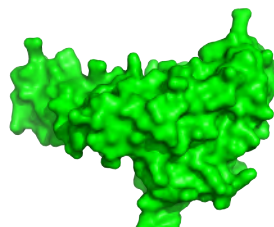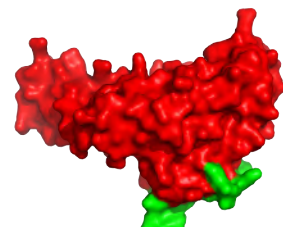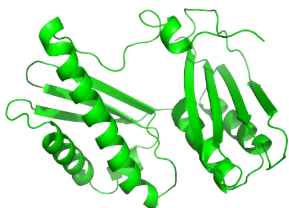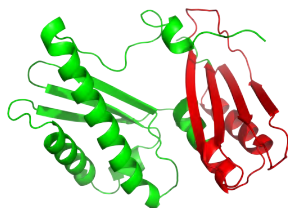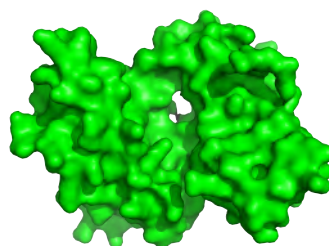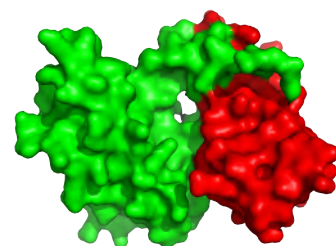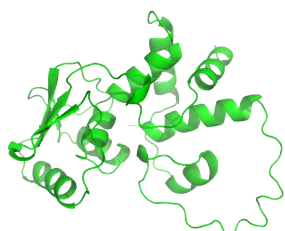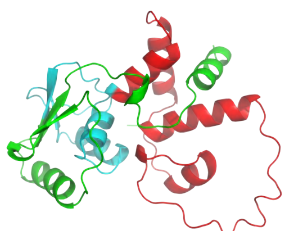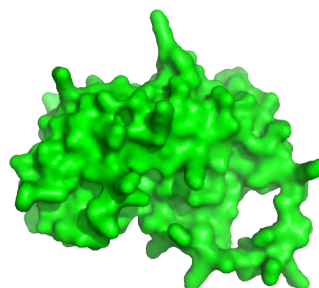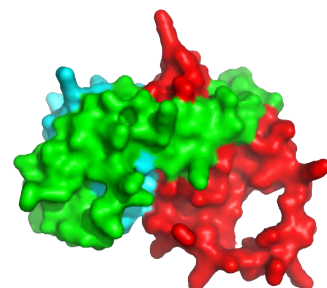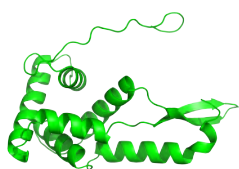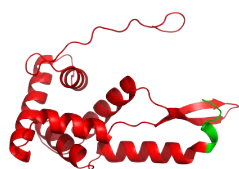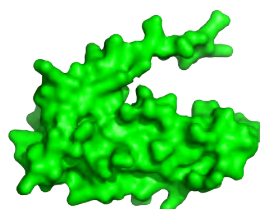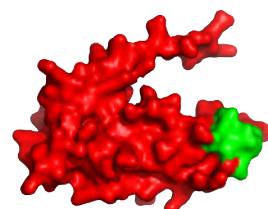

Supplement: Supplementary file 6 [file DataSheet_8.pdf]

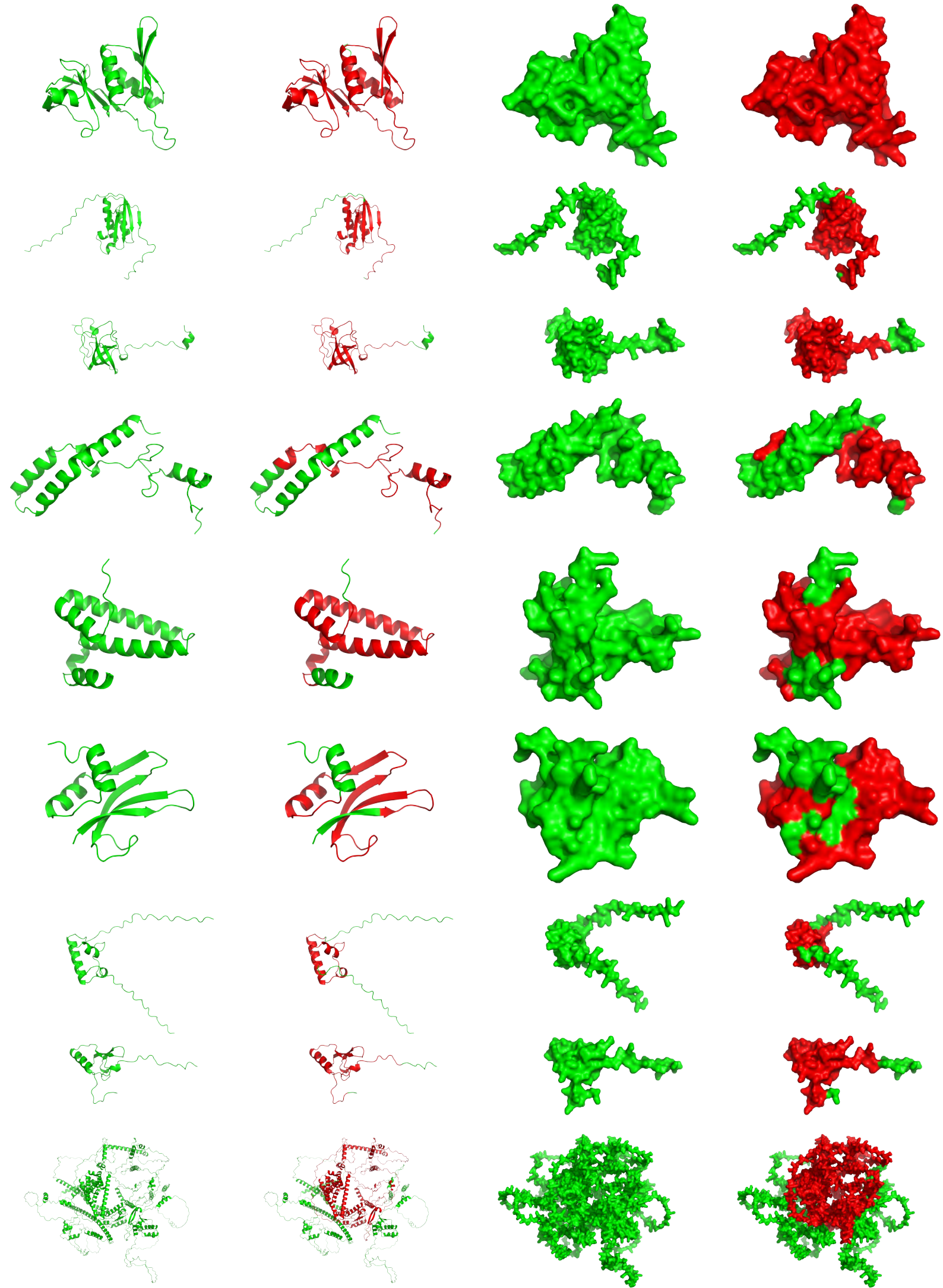

Supplement: Supplementary file 7 [file DataSheet_9.pdf]

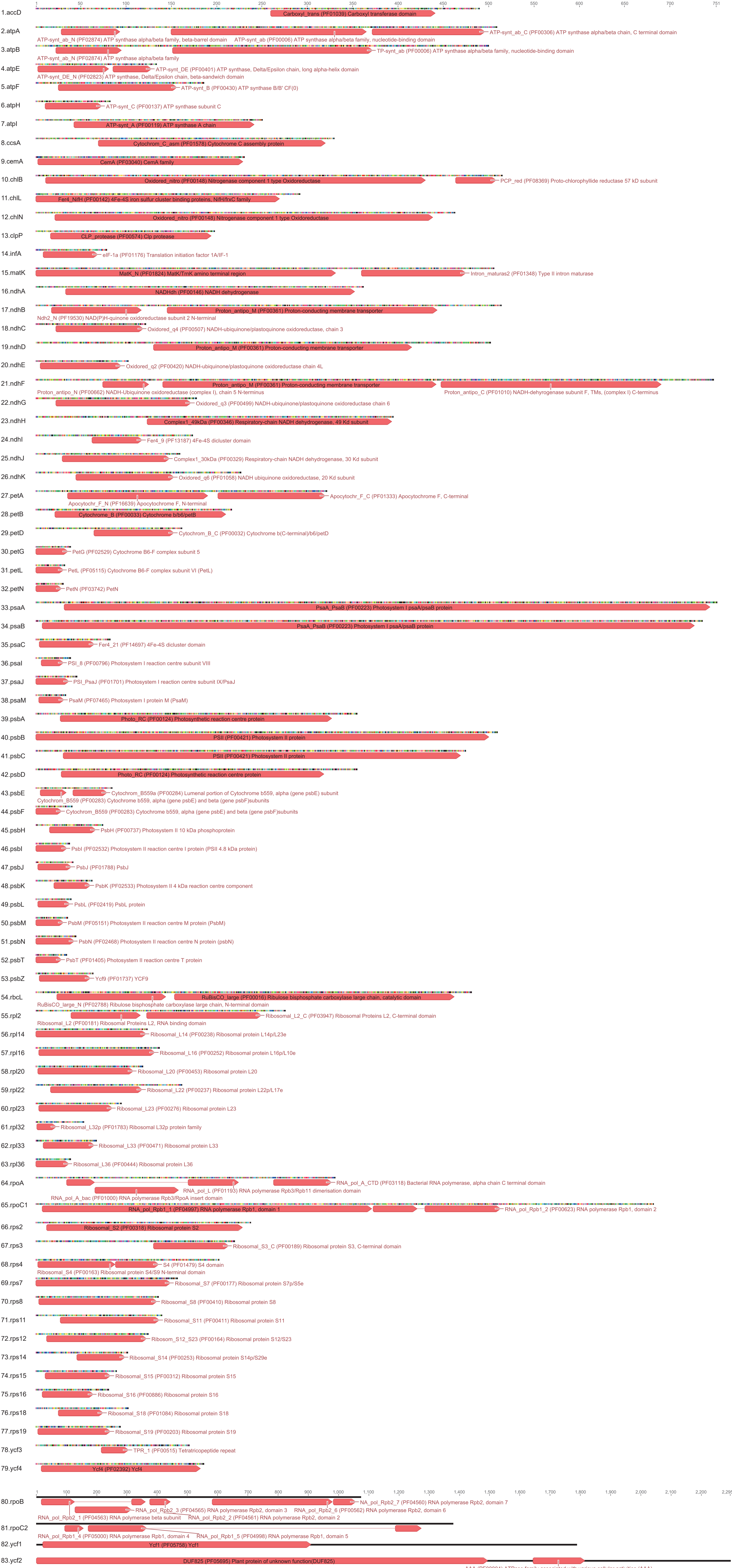

Supplement: Supplementary Figure 1 — The inferred functional domains for all PCGs occurred in seed plants, for infA in Amborella trichopoda, for chlB/L/N and psaM in Zamia furfuracea, and for remaining PCGs in Arabidopsis thaliana. The domain names and their coordinates are shown, with 1st to 79th PCGs sharing a coordinate and with 80th to 83th PCGs sharing another coordinate. [file DataSheet_1.pdf]

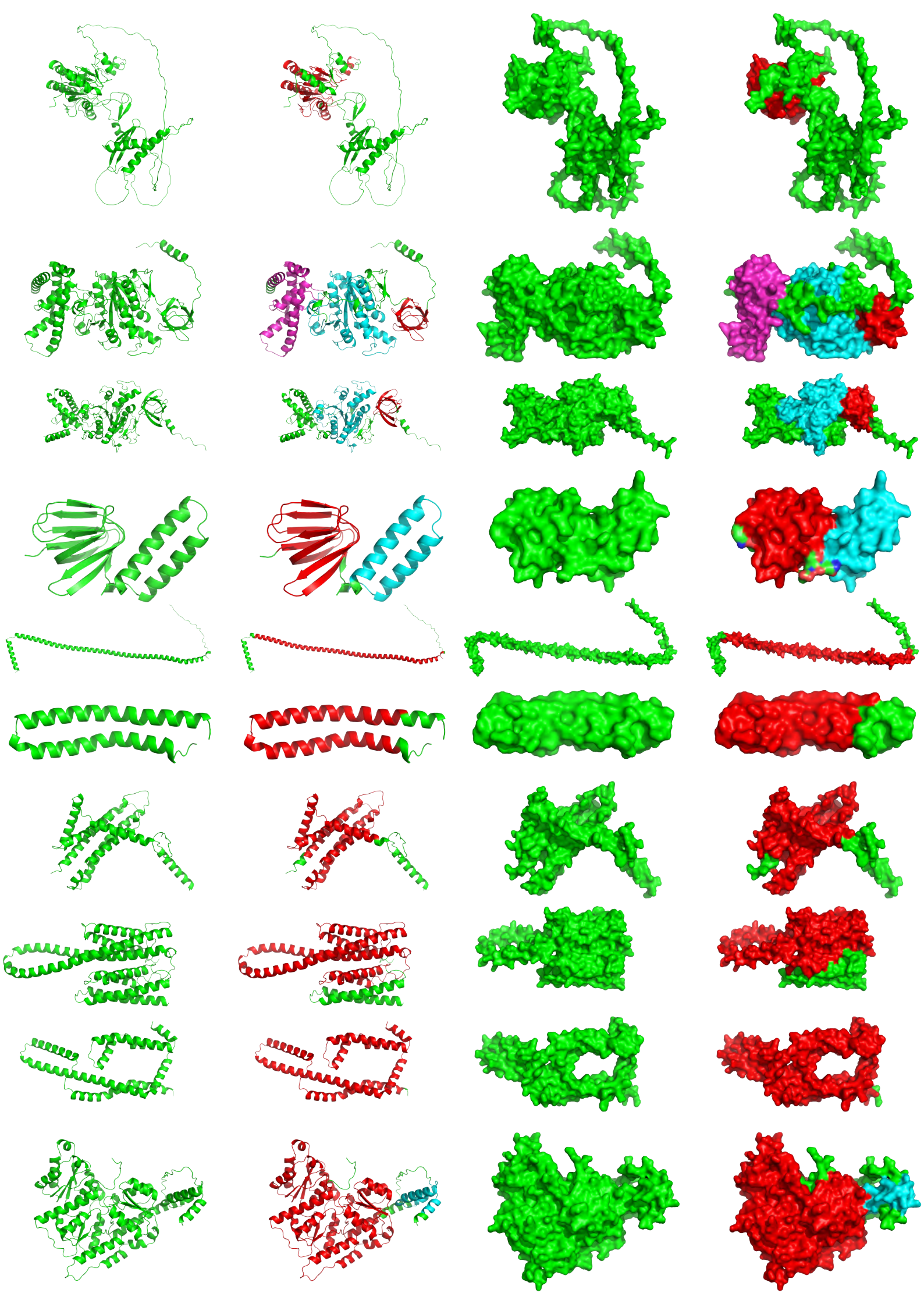

Supplement: Supplementary Figure 2 — The AlphaFold-predicted protein structures for all PCGs occurred in seed plants, for infA in Amborella trichopoda, for chlB/L/N and psaM in Zamia furfuracea, and for remaining PCGs in Arabidopsis thaliana. The protein structures are sorted according to the gene names in Table S1 . The first column indicates the predicted protein structure in cartoon form, with the protein structure shown as green color. The second column indicates the predicted protein structure in cartoon form, with the domain shown as red color and the remaining residues shown as green color. The third column indicates the predicted protein structure in surface form, with the protein structure shown as green color. The fourth column indicates the predicted protein structure in surface form, with the domain shown as red color and the remaining residues shown as green color. [file DataSheet_2.pdf]
